# Plasmonic- and Electronic-Enhancement-Free Coherent Raman Detection of Ångström-Scale Molecular Layers at Metal Interfaces

**DOI:** 10.1021/acs.nanolett.6c00802

**Published:** 2026-04-27

**Authors:** Toshiki Sugimoto, Tomoaki Ichii, Tsuneto Kanai, Ryu Yoshizawa, Shota Takahashi, Atsunori Sakurai, Keisuke Seto, Chengxiang Jin

**Affiliations:** † 88301 Institute for Molecular Science, National Institutes of Natural Sciences, Okazaki, Aichi 444-8585, Japan; ‡ Graduate Institute for Advanced Studies, SOKENDAI, Okazaki, Aichi 444-8585, Japan; § Laser-driven Electron-acceleration Technology Group, RIKEN Spring-8 Center, Sayocho, Hyogo 679-5148, Japan

**Keywords:** interfacial Raman spectroscopy, coherent Raman spectroscopy, nonlinear spectroscopy, molecular vibration, metal interfaces

## Abstract

Coherent Raman scattering provides highly sensitive vibrational analysis through nonlinear light-matter interactions. However, its application to metal interfaces remains challenging because the intrinsically large nonresonant background (NRB) of metals overwhelms weak interfacial molecular vibrational signals. Here, we report a time-frequency hybrid coherent Raman spectroscopy approach that overcomes this limitation and enables the sensitive detection of ångström-thick molecular systems even on atomically flat metal surfaces. Our method combines a femtosecond pump and Stokes pulses with a time-delayed, asymmetrically shaped picosecond probe pulse. By exploiting the instantaneous temporal response of the metal NRB, this scheme effectively filters out the dominant NRB while retaining a controlled residual NRB that acts as an internal local oscillator, enabling the interferometric amplification of weak interfacial vibrational signatures. This all-optical coherent enhancement strategy establishes a route for direct, noninvasive Raman detection of interfacial molecular systems across diverse surfaces without requiring structure- and material-specific plasmonic and electronic enhancement mechanisms.

## Graphical abstract

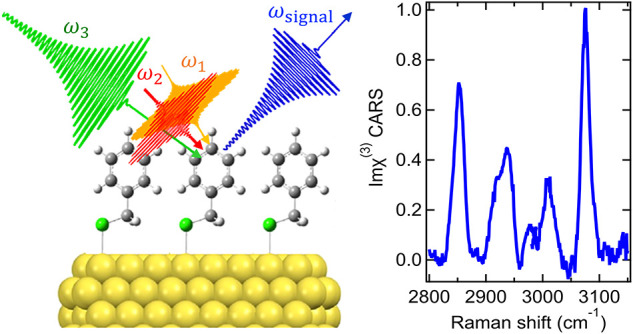

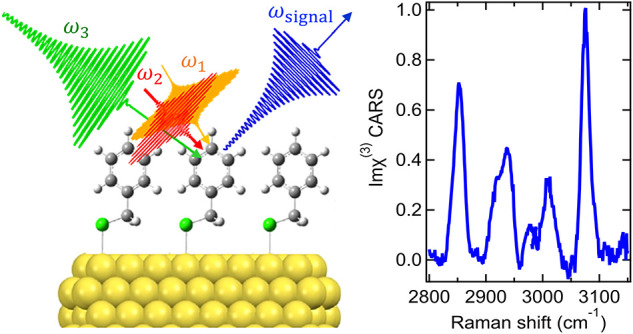

Molecular vibrations provide a direct fingerprint of chemical bonding, structure, and intermolecular interactions, making vibrational spectroscopy an indispensable tool across chemistry, physics, and materials science.^1–8^ Raman spectroscopy, which probes vibrations through inelastic light scattering, has therefore become a cornerstone technique because it accesses infrared-inactive vibration, operates under ambient conditions, and offers broad chemical specificity for complex systems.^1,9–12^ Despite these advantages, extending Raman spectroscopy to molecular systems confined at surfaces and interfaces remains fundamentally challenging, due to the extremely small number of molecules involved and intrinsically weak cross section of spontaneous Raman scattering.^13–18^

For interfacial molecular layers with thickness ranging from a few nanometers down to the ångström scale, spontaneous Raman measurements typically require high excitation intensities, long acquisition times, and stringent experimental constraints to suppress fluorescence, while often yielding limited signal-to-noise ratios.^13–16^ To overcome these limitations, a wide range of enhancement strategies have been developed, including electronic resonance Raman spectroscopy^2,11,19^ and plasmonic field enhancement approaches such as surface-enhanced Raman spectroscopy (SERS),^11,17,18,20^ shell-isolated nanoparticle-enhanced Raman spectroscopy (SHINERS),^11,21,22^ and tip-enhanced Raman spectroscopy (TERS).^2,11,18^ ^,^ ^23–25^ In some cases, charge-transfer interactions at metal-molecule interfaces provide an additional contribution to chemical enhancement.^11,17,18,26^ While these approaches have enabled remarkable sensitivity gains and advanced interfacial Raman spectroscopy, their applicability and reliability often depend on specific nanostructures, materials, or molecular systems, and they may perturb interfacial molecular environment under investigation.^25,27^ This has stimulated interest in complementary Raman methodologies that aim to achieve high sensitivity without relying on these enhancement strategies.

Coherent Raman scattering techniques, such as coherent anti-Stokes Raman scattering (CARS), offer an alternative route to highly sensitive vibrational spectroscopy by exploiting stimulated third-order nonlinear light-matter interactions.^1,28–32^ In CARS, Raman active molecular vibrations with a frequency *Ω*_0_ are coherently driven by the frequency difference between pump (*ω*_1_) and Stokes (*ω*_2_) fields (*ω*_1_ – *ω*_2_ ≡ *Ω* = *Ω*_0_,), and the resulting vibrational coherence is up-converted by a probe pulse (*ω*_3_) into an anti-Stokes signal at *ω*_AS_ = *ω*_1_ – *ω*_2_ + *ω*_3_ through a four-wave mixing process. The coherent nature of this process typically yields directional signals that can be orders of magnitude stronger than spontaneous Raman scattering.^28–32^ However, applying coherent Raman spectroscopy to interfacial molecular systems remains challenging because strong vibrationally nonresonant background (NRB) signals^32,33^ arising from adjacent bulk media or substrates often overwhelm the weak vibrational response from interfaces. Various suppression strategies of the dominant NRB, such as polarization control detection,^34^ interferometric detection,^35^ and heterodyne detection,^36,37^ have been developed to mitigate this problem, yet they remain insufficient for reliably detecting vibrational signals from interfacial layers.

For transparent dielectric substrates, where the vibrationally nonresonant third-order nonlinear susceptibility is typically modest,^28,38,39^ specific reflection-based geometries and polarization schemes near the Brewster angle have been shown to suppress NRB contributions and enable coherent Raman detection of interfacial molecular layers.^33,40,41^ In contrast, metal substrates pose a fundamentally more severe challenge. Owing to their strong optical reflectivity and large nonresonant third-order nonlinear susceptibility,^28,39^ the intense NRB generated by metals cannot be effectively mitigated by optical geometry, preventing straightforward extension of coherent Raman techniques to metal-supported molecular layers.^42,43^ As a practical consequence, coherent Raman spectroscopy of interfacial molecular systems on metal has often relied on plasmonic nanostructures,^18^ thereby limiting generality and quantitative applicability.

In this work, we demonstrate a CARS spectroscopy approach that overcomes these limitations and enables sensitive detection of ångström-thick molecular layers on flat metal surfaces. Our method employs a hybrid time-frequency detection scheme,^32,33,44–49^ in which femtosecond pump and Stokes pulses generate vibrational coherence, while a time-delayed, asymmetrically shaped picosecond probe pulse with a sharp femtosecond-scale rising edge selectively samples the nonlinear vibrational response^50–53^ (Figure 1). By exploiting the inherently instantaneous nature of the temporal response of the metal NRB, this engineered pulse configuration effectively filters out the dominant background in the time domain, achieving more than 4 orders of magnitude suppression. Then, rather than eliminating the NRB entirely, the controlled residual NRB is harnessed as an internal local oscillator, enabling the coherent interferometric amplification of weak interfacial vibrational signals. This approach provides a direct and noninvasive route to highly sensitive Raman detection of ultrathin molecular layers even on atomically flat nonplasmonic metal substrates under electronically nonresonant conditions.

**Figure 1:**
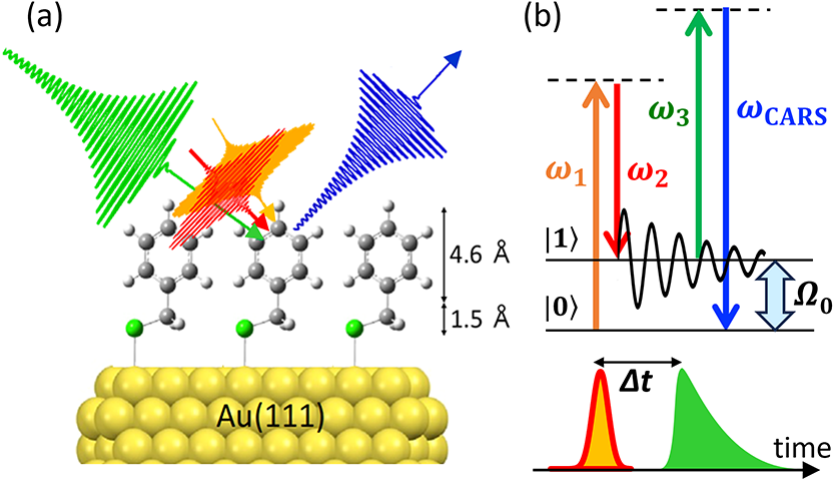
(a) Schematic experimental geometry of the time-delayed femtosecond/picosecond hybrid three-color CARS scheme applied to an ångström-scale self-assembled monolayer on an atomically flat Au(111) substrate. (b) Energy-level diagram and temporal pulse sequence of the time-delayed three-field CARS scheme. For visual clarity, different colors are used in the schematic to distinguish the independently prepared probe field (ω_3_) from the pump and Stokes excitation fields (ω_1_ and ω_2_).

As a model system for metal-supported ångström-thick molecular layers free from plasmonic and electronic resonance enhancement, a self-assembled monolayer (SAM) of benzyl mercaptan (BM, ∼ 10^14^ molecules cm^–2^)^54–56^ was prepared on an atomically flat Au(111) surface (see Supporting Information Section 1 for preparation details). BM-SAM provides a structurally simple and chemically well-characterized interface, consisting of a quasi-centrosymmetric phenyl ring (∼ 5 Å thick) and an anti-centrosymmetric methylene unit (∼ 2 Å thick), as illustrated in Figure 1(a). This structural motif naturally incorporates vibrational modes that either obey or violate the infrared-Raman mutual exclusion rule, offering an ideal platform for assessing the intrinsic sensitivity of the present CARS approach to Raman-active interfacial vibrations without relying on infrared activity.

All measurements were performed by using near-infrared excitation, well-away from the electronic resonances of both the molecular layer and the Au(111) substrate (below 540 nm). A three-field CARS scheme was implemented using a femtosecond Yb:KGW laser source (CAERBIDE, Light Conversion) operating at a repetition rate of 200 kHz, generating femtosecond pump (ω_1_, λ_1_ = 1034 nm, fwhm ≈ 8.5 nm, 400 nJ) and tunable broadband (ω_2_, λ_2_ = 1150–1500 nm, fwhm ≈ 80 nm, 100 nJ) Stokes pulses, together with a time-delayed, asymmetrically shaped picosecond narrowband probe pulse (ω_3_, λ_3_ = 1034 nm, fwhm ≈ 0.7 nm (6.3 cm^–1^), 2.7 μJ). The probe pulse featured a sharp femtosecond rising edge, followed by a picosecond decay (Figure S1), enabling selective temporal sampling of the vibrational response. These three beams were spatially overlapped on the sample in reflection geometry with *p*-polarized excitation. In this configuration, the detected CARS signal in the *pppp* polarization combination is dominated by the *zzzz* component of the third-order nonlinear susceptibility tensor, χ^(3)^_zzzz_, where *z* is defined as the surface-normal direction. This dominance arises from the Fresnel local field factor at the metal surface.^17^ No detectable thermal effects or laser-induced damage were observed under ambient conditions, confirming that the measurements were performed in a noninvasive regime under the present irradiation conditions (see Supporting Information Section 1 and Figure S9 for experimental details).

To rationalize the design principle of the time-frequency hybrid three-field CARS approach, it is essential to consider the distinct temporal characteristics of vibrationally nonresonant and resonant nonlinear responses at metal-supported interfacial molecular systems. As established in previous studies^28–37,44–53^ the NRB originates from an instantaneous four-wave-mixing response and is therefore governed by the temporal overlap of the of the pump, Stokes, and probe pulses (Supporting Information Section 3), whereas the vibrationally resonant CARS signal is governed by the free induction decay of molecular coherence persisting over the vibrational dephasing time *T*_2_ (Supporting Information Section 4). Notably, for ultrathin molecular layers adsorbed on metal substrates, as considered here, the vibrationally resonant CARS signal arises from interfacial molecules, whereas the NRB originates from a metal-induced four-wave-mixing response within the penetration depth of the optical fields, which is typically on the order of ∼ 10 nm in a reflection geometry.^28^ In conventional two-field CARS schemes, one of the excitation fields (pump or Stokes) is simultaneously used as the probe pulse, which inevitably leads to complete temporal overlap of the pump, Stokes, and probe pulses and inevitably generates a strong NRB, which often overwhelms weak vibrational responses from interfacial molecular layers. While multifield CARS schemes have been developed to mitigate this limitation,^32,33,44–49^ they typically employ temporally symmetric or sinc-shaped picosecond probe pulses, for which substantial NRB suppression generally requires longer delay times (Figure S3). This can be less favorable for metal-supported molecular layers, where the NRB is exceptionally large, and for systems with rapid vibrational dephasing. In contrast, the present three-field CARS configuration employs an independently prepared probe pulse with a time-asymmetric temporal envelope combining a femtosecond-scale sharp rising edge and a picosecond-scale duration (Figure S1). This design enables precise control of pulse overlap and a rapid reduction of the NRB over delays in the femtosecond to sub-picosecond regime, while preserving sensitivity to the vibrational free induction decay (Figure 1(b)). As a result, frequency-domain detection of the nonlinear optical response can be achieved with high sensitivity to resonant vibrational contributions on ultrafast time scales (see also Supporting Information Sections 3 and 4).

Figure 2(a) shows the time-delayed CARS intensity spectra, *I*_CARS_(*Ω*; *Δt*), in the C–H stretching region as a function of the Raman shift (*Ω* ≡ *ω*_1_ – *ω*_2_) and probe delay *Δt*. At short probe delays (*Δt* ≲ 300 fs), the vibrationally resonant features are completely obscured by an intense NRB, *I*_CARS_(*Ω*; *Δt*) ≈ *I*_NRB_(*Ω*; *Δt*) ∝ |*P̃*_NR_^(3)^(*Ω*; *Δt*)|^2^, where *P̃*_NR_^(3)^(*Ω*; *Δt*) denotes the frequency-domain vibrationally nonresonant third-order polarization originating from the gold substrate. It should be noted that although the detected signal corresponds to the intensity of the radiated third-order field |*E* ^(3)^|^2^, the nonlinear response in the present study is described in terms of the corresponding third-order polarization, following the standard convention in CARS spectroscopy^28–37^ ^,^ ^44–53^ based on the relation *E* ^(3)^ ∝ *P* ^(3)^. Because the vibrationally nonresonant third-order nonlinear susceptibility χ_NR_^(3)^ is typically frequency-independent,^28,31,32^ ^,^ ^46–49^ the NRB exhibits a broadband, featureless spectral profile determined by the convolution of the spectral distributions of the pump and Stokes pulses (eq S9). The decay profile of the NRB intensity with increasing probe delay *Δt* is shown in Figure 2(b) and is well-described by numerical simulations that account for the asymmetric temporal profile of the probe pulse (Figure S3). As the probe delay *Δt* increases, the NRB intensity is progressively suppressed by more than 4 orders of magnitude relative to that at zero probe delay (*Δt* = 0 fs). Even at delays where the main pulse peaks are largely separated, partial overlap of the lower-intensity pulse tails produces much weakened but finite NRB signal (see also Supporting Information Section 3 for details).

**Figure 2:**
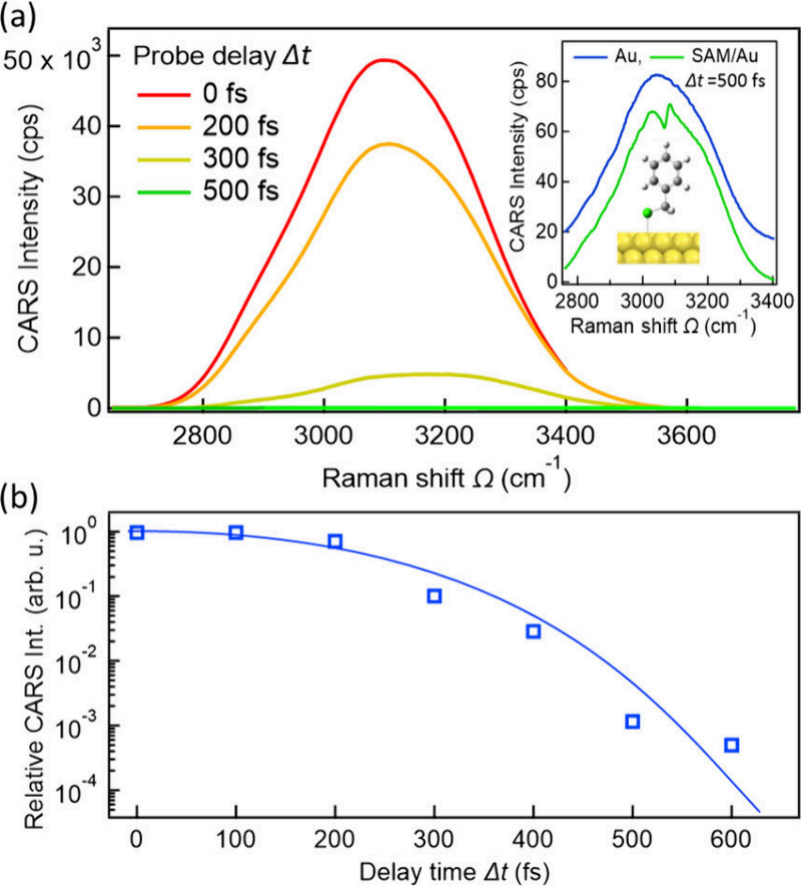
(a) Time-delayed CARS intensity spectra measured from Au(111) substrate covered with a benzyl mercaptan (BM) self-assembled monolayer (SAM) at various probe delay time *Δt*. Inset: CARS intensity spectrum at *Δt* = 500 fs (green) is compared with that obtained from bare Au(111) under identical optical conditions (blue). These spectra are vertically offset for visual clarity. (b) Probe-delay-time dependence of the vibrationally nonresonant background (NRB) intensity, normalized to the value at *Δt* = 0 fs. The solid curve shows the simulated *Δt* dependence of the NRB based on the temporal overlap of the pump, Stokes, and probe pulses (eqs S7 and S15, Figure S3­(c), see also Supporting Information Section 3).

As shown in the inset of Figure 2(a), a pronounced dip-like feature emerged in the broadband NRB spectrum at *Δt* = 500 fs. In contrast, this spectral feature is absent for a pristine Au(111) surface without molecular adsorption (inset of Figure 2(a)), confirming its molecular vibrational origin. The dip at ∼ 3070 cm^–1^ is very close to the resonant frequency of the highly symmetric phenyl C–H stretching modes that are Raman-active but infrared-inactive (Figures 4 and S10). Notably, this dip feature originates from the coherent superposition in the frequency domain between the radiated field generated by the vibrationally resonant third-order polarization, *P̃*_R_^(3)^(Ω; *Δt*), and that generated by the nonresonant polarization, *P̃*_NR_^(3)^(Ω; *Δt*). Under these conditions, the nonresonant contribution remains a few orders of magnitude larger than the resonant one (i.e., |*P̃*_NR_^(3)^| ≫ |*P̃*_R_^(3)^|), so that the vibrational CARS signal is predominantly encoded in their interference term:

ICARS(Ω;Δt)∝|P∼NR(3)(Ω;Δt)+P∼R(3)(Ω;Δt)|2≈|P∼NR(3)(Ω;Δt)|2+2Re[P∼NR(3)*(Ω;Δt)P∼R(3)(Ω;Δt)] 1

while the purely resonant term |*P̃*_R_^(3)^|^2^ is negligibly small. When the temporal width of the pump and Stokes pulses and the leading edge of the probe pulse are much shorter than the vibrational dephasing time *T*_2_, and the probe bandwidth is narrower than the vibrational line width, the resonant polarization can be expressed as

$${\overset{∼}{P}}_{R}^{\left(3\right)}\left(\Omega ;\Delta t\right)={\overset{∼}{P}}_{R0}^{\left(3\right)}\exp\left(-\Delta t/T_{2}\right)\chi_{R}^{\left(3\right)}\left(\Omega \right)$$ 2

where χ_*R*_^(3)^(Ω) = *A*_0_/(Ω_0_ – Ω – *i*/*T*_2_) represents the vibrational resonant contribution to the third-order nonlinear susceptibility (eq S26). This expression connects the time-domain decay of molecular coherence to the frequency-domain vibrational line shape (Figure S4). Substituting eq 2 into eq 1 and introducing ϕ_*NR*_ as the phase of *P̃*_NR_^(3)^ gives

ICARS(Ω;Δt)≈|P∼NR(3)(Ω;Δt)|2+2|P∼NR(3)(Ω;Δt)|×P∼R0(3)exp(−Δt/T2)Re[e−iϕNRχR(3)(Ω)] 3

Normalizing the total CARS intensity *I*_CARS_(Ω; *Δt*) by the pure NRB intensity *I*_NRB_(Ω; *Δt*) ∝ |*P̃*_NR_^(3)^(Ω; *Δt*)|^2^ yields a simple form for the vibrational response:

ICARS(Ω;Δt)INRB(Ω;Δt)≈1+S(Δt)Re[e−iϕNRχR(3)(Ω)] 4

where *S*(*Δt*) ≡ 2*P̃*_*R*0_^(3)^ exp (−*Δt*/*T*_2_)/|*P̃*_NR_^(3)^(Ω; *Δt*)| (eq (S32)). The spectral shape of the normalized CARS intensity is thus governed by the term Re­[*e* ^–*iϕ*_*NR*_^χ_*R*_^(3)^(Ω)]. When ϕ_*NR*_ = 0 or π, the vibrational response of the normalized CARS intensity follows Reχ_*R*_^(3)^(Ω), resulting in a dispersive (derivative-like) line shape (Figure S6­(a)). In contrast, when ϕ_*NR*_ = ± π/2, the normalized CARS spectrum reflects Imχ_*R*_^(3)^(Ω), yielding an absorptive peak (Figure S6­(b)).

Figure 3(a) shows the normalized CARS spectrum obtained by dividing the measured CARS intensity spectrum by the NRB intensity spectrum at a probe delay of *Δt* = 500 fs, based on the data shown in the inset of Figure 2(a). The normalized spectrum exhibits a pronounced derivative-like feature at around ∼ 3070 cm^–1^, together with a weaker dispersive vibrational response near 3010 cm^–1^ that originates from less-symmetric phenyl C–H stretching modes that are both Raman- and infrared-active (Figure S10). As shown in Figure S7­(c), these spectral features can be qualitatively reproduced by fitting with the two Lorentzian components χ_*R*_^(3)^(Ω) = ∑_*k*=1,2_ *A*_0*k*_/(Ω_0*k*_ – Ω – *i*/*T*_2*k*_) with a nonresonant phase of ϕ_*NR*_ ≈ π (Figure S7­(c)). This analysis indicates that the vibrational contribution in the normalized CARS spectrum (Figure 3(a)) predominantly reflects the real part of χ_*R*_^(3)^(Ω).

**Figure 3:**
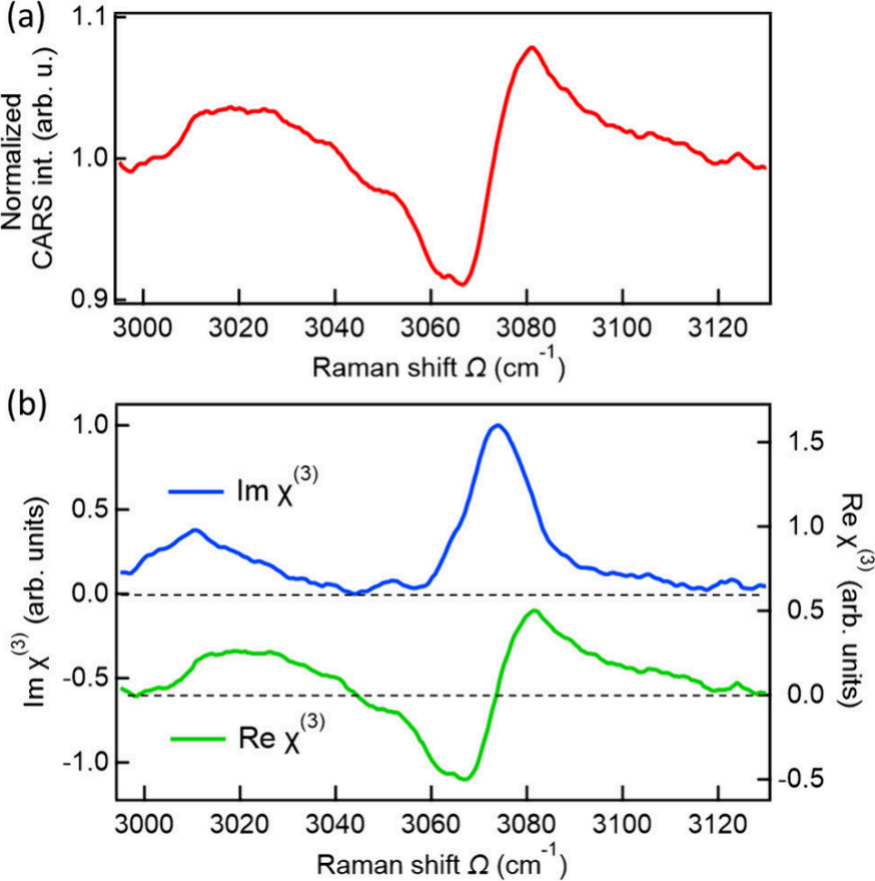
(a) Normalized CARS intensity spectrum measured at *Δt* = 500 fs in the C–H stretching region of the phenyl group of a BM-SAM on Au(111). (b) Imχ_*R*_^(3)^(Ω) spectrum (blue, left axis) and Reχ_*R*_^(3)^(Ω) spectrum (green, right axis) retrieved by the maximum entropy method from the normalized CARS intensity spectrum shown in (a).

To further retrieve accurate spectral line shapes without assuming any prior model, the maximum entropy method (MEM)^31^ was applied to the normalized CARS spectrum (Figure 3(a)). This approach successfully reconstructs both the real and imaginary components of χ_*R*_^(3)^(Ω), providing a model-independent characterization of the interfacial vibrational response. The reconstructed Reχ_*R*_^(3)^(Ω) and Imχ_*R*_^(3)^(Ω) spectra obtained by the MEM analysis are shown in Figure 3(b). In the Imχ_*R*_^(3)^(Ω) spectrum, clear vibrational peaks are observed at Ω ∼3075 cm^–1^ and Ω ∼3010 cm^–1^. Importantly, the Imχ_*R*_^(3)^(Ω) spectrum directly corresponds to the line shape of the spontaneous Raman spectrum.^31^ Therefore, the successful detection of these vibrational modes, arising from a molecular layer with ångström-scale thickness, clearly demonstrates the sensitivity of our coherent Raman spectroscopic approach for probing metal-supported ultrathin interfacial molecular systems without relying on plasmonic, electronic, and chemical enhancement mechanisms.^18^

By tuning the center wavelength of the ω_2_ Stokes pulse between 1150 and 1500 nm and performing similar time-delayed CARS measurements combined with MEM analyses at *Δt* = 500 fs, we obtained the Imχ_*R*_^(3)^(Ω) and Reχ_*R*_^(3)^(Ω) spectra covering both the fingerprint and C–H stretching regions (Figure 4)­. The Imχ_*R*_^(3)^(Ω) spectra exhibit multiple distinct vibrational resonances (A-K), which can be classified into Raman-active but infrared-inactive modes (A-D, I, and K) and modes that are active in both Raman scattering and infrared absorption (E-H and J). As also shown in Figure S10 in more detail, the Raman-active but infrared-inactive modes include highly symmetric phenyl-ring vibrations such as the ring-breathing (A), ring-bending (B), ring-bending coupled with CH_2_ twisting (C), multiple ring-bending coupled with CH_2_ twisting or wagging (D), phenyl C–H stretching vibration with minor ring-deformation character (I), and symmetric phenyl C–H stretching (K) modes. In contrast, Raman- and infrared-active modes are assigned as follows: (E) CH_2_ wagging, (F) CH_2_ symmetric C–H stretching involved in Fermi resonance with a CH_2_ bending overtone, (G) phenyl C–H vibration coupled with ring-bending motion, (H) CH_2_ bending overtone involved in Fermi resonance with the symmetric CH_2_ C–H stretching vibration, and (J) multiple lower-symmetry phenyl C–H stretching modes.^55,56^

**Figure 4:**
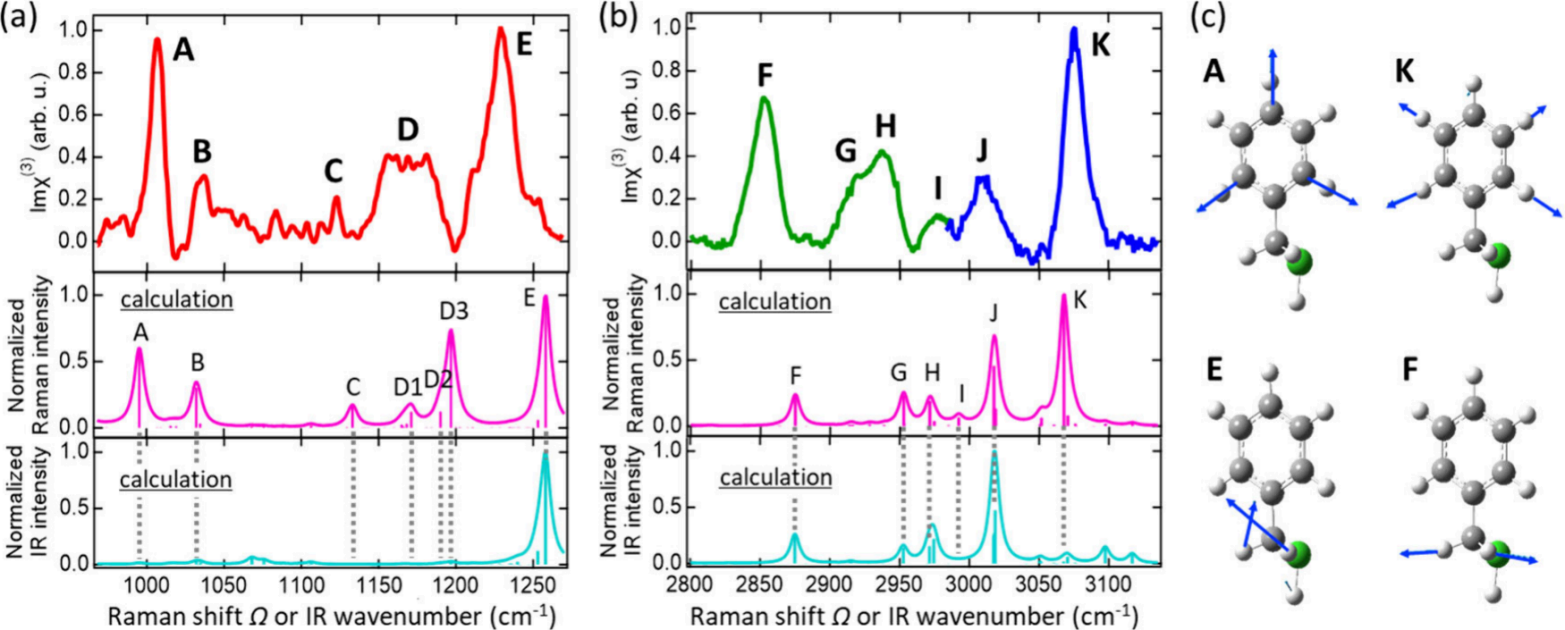
Vibrational spectra of the BM-SAM on Au(111) in (a) the fingerprint region and (b) the C–H stretching region. Top: experimentally retrieved Imχ_*R*_^(3)^(Ω) spectra. Middle and bottom: Raman intensity and IR absorption spectra of a BM molecule simulated by DFT calculation. (c) Representative vibrational motion of Raman-active but IR-inactive modes (A, K) of the quasi-centrosymmetric phenyl ring and of modes that are both Raman- and IR-active (E, F) for the anticentrosymmetric methylene group (see Methods and Figure S10 for more details).

The assignments are based on anharmonic vibrational calculations for an isolated BM molecule (see Methods for calculation details), which are used here as a reference for spectral interpretation rather than as a fully quantitative model of all experimental peak intensities. Compared with previous harmonic calculations,^55,56^ the present anharmonic treatment describes characteristic features such as the Fermi-resonance-related peaks in the C–H stretching region more appropriately. The relatively enhanced experimental intensities of modes F and H, compared with the calculated Raman intensities for an isolated BM molecule (middle of Figure 4(b)), mainly involve C–H stretching vibrations of the CH_2_ group located closer to the gold surface and may be partially affected by adsorption-induced, mode-dependent chemical enhancement of the Raman response^26^ at the gold interface.

This systematic assignment reveals that the present interfacial CARS approach enables comprehensive probing of vibrational modes spanning a wide range of molecular symmetries from highly symmetric Raman-dominant modes to lower-symmetry modes exhibiting mixed Raman and infrared activity. We note that similar time-delayed CARS measurements have also been performed for other ultrathin molecular layers, and the corresponding Imχ_*R*_^(3)^(Ω) spectra are presented in Figure S11, indicating that the present time-frequency hybrid CARS approach is not restricted to the BM-SAM system but is applicable to other interfacial molecular layers without relying on plasmonic and electronic enhancement.

On the basis of these demonstrations of interfacial CARS molecular spectroscopy, we now address a fundamental question: whether coherent Raman techniques genuinely offer a sensitivity advantage over spontaneous Raman scattering for ångström-scale ultrathin interfacial systems, in contrast to bulk samples with micrometer-scale thickness.^29,30^ From a general perspective, spontaneous Raman scattering is intrinsically incoherent, and its signal scales linearly with the number of molecules *N*, while the intensity of CARS signals, derived from the purely resonant third-order polarization, scale as |*P̃*_R_^(3)^|^2^ ∝ *N* ^2^. This quadratic scaling has led to the prevailing assumption that the intrinsic signal advantage of coherent Raman techniques diminishes in the ultrathin limit, particularly for molecular layers with ångström-scale thicknesses. Under a pulse irradiation condition constrained by the damage threshold of the BM-SAM on Au (Figure S9 and Supporting Information Section 1), prior theoretical analyses^29,30^ indicate that the time-averaged total photon flux generated by purely resonant CARS is comparable to, but typically a few times larger than, that of spontaneous Raman scattering for ångström-scale interfacial layers.

At this stage, however, it is crucial to note that this comparison includes only the purely resonant contribution |*P̃*_R_^(3)^(Ω; *Δt*)|^2^. In the present interfacial CARS measurements on metal substrates, an additional interference contribution can also arise: the residual NRB signal from the metal substrate (inset of Figure 2(a)) serves as an intrinsic local oscillator^8,46,47,50^ that coherently amplifies the weak interfacial vibrational response through optical interference. The vibrationally resonant component *P̃*_R_^(3)^(Ω; *Δt*), directly related to χ_*R*_^(3)^(Ω) (eq (S26)), appears in the second term of eq 1. Since the interference term in eq 1 can be expressed as 2|*P̃*_NR_^(3)^(Ω; *Δt*)||*P̃*_R_^(3)^(Ω; *Δt*)| cos ΔΦ, where ΔΦ is phase difference between *P̃*_R_^(3)^(Ω; *Δt*) and *P̃*_NR_^(3)^(Ω; *Δt*), we define a coherent amplification factor, *w*_R_, as the ratio of the amplified resonant term 2|*P̃*_NR_^(3)^(Ω; *Δt*)||*P̃*_R_^(3)^(Ω; *Δt*)| to the intrinsic resonant term |*P̃*_R_^(3)^(Ω; *Δt*)|^2^ in the absence of NRB at Ω = Ω_0_, *w*_R_ ≡ 2|*P̃*_NR_^(3)^(Ω_0_; *Δt*)||*P̃*_R_^(3)^(Ω_0_; *Δt*)|/|*P̃*_R_^(3)^(Ω_0_; *Δt*)|^2^. This can be equivalently rewritten as *w*_R_ = 2|*P̃*_NR_^(3)^(Ω_0_; *Δt*)|^2^/|*P̃*_NR_^(3)^(Ω_0_; *Δt*)||*P̃*_R_^(3)^(Ω_0_; *Δt*)| and experimentally estimated to be *w*_R_ ≈ 30 in our case (*Δt* ≈ 500 fs, inset of Figure 2(a); see also Supporting Information Section 7 for more details of this favorable amplification condition). Combining this interferometric amplification factor with the above estimate that the total photon flux generated by purely resonant CARS is a few times larger than that of spontaneous Raman scattering, the effective signal-generation efficiency of this interferometrically amplified interfacial CARS approach is expected to exceed that of spontaneous Raman scattering by approximately 2 orders of magnitude.

In addition to this signal-generation advantage, the overall sensitivity balance is further enhanced by the signal-detection efficiency. Whereas spontaneous Raman photons are emitted isotropically and collected over a limited solid angle (typically a few %), coherent Raman signals are emitted directionally and collected with near-unity efficiency. Taking this signal directionality and the resulting collection efficiency into account, the experimentally detectable time-averaged photon flux of the present interfacial CARS approach is estimated to exceed that of spontaneous Raman scattering by approximately 4 orders of magnitude. In this context, we note that spontaneous Raman measurements performed under continuous-wave irradiation at 532 and 633 nm, using optical configurations and acquisition times comparable to those of the present CARS experiments, yielded no detectable signal from the BM-SAM on Au(111). This observation is consistent with earlier reports in which unenhanced spontaneous Raman signals from sub-nanometer adsorption layers on flat metal surfaces were only marginally detectable despite substantially higher probe intensities and significantly longer acquisition times.^13–17^

Establishing a sensitivity advantage of interfacial CARS over spontaneous Raman scattering, we note that its practical implementation at metal interfaces requires careful control of the laser parameters. Molecular layers on conductive substrates exhibit relatively low laser damage thresholds (Figure S9) compared with transparent liquids and dielectric materials.^33,40,41^ Consequently, conventional low-repetition-rate (10 Hz – 1 kHz), high-pulse-energy laser sources (mJ-class)^33,40,41^ are unsuitable for metal-supported ultrathin molecular layers. In this case, simply attenuating such high-energy pulses is not an effective solution because the third-order nonlinear polarization underlying CARS scales with the cube of the optical electric field; reducing the pulse energy to avoid damage therefore leads to a significant loss of signal that cannot be compensated by averaging at low repetition rates. At the opposite extreme, high-repetition-rate (∼100 MHz) oscillator-based light sources (nJ-class)^18^ are also fundamentally unsuitable because their weak pulse electric fields are typically too weak to generate measurable unenhanced interfacial CARS signals, even after averaging over practical acquisition times. Enabled by recent advances in Yb-based laser technology,^52^ the intermediate repetition-rate regime adopted here (100 kHz–1 MHz) with pulse energies of 0.1–1 μJ at the sample therefore provides a practical and robust solution.

Within this experimentally accessible optical parameter regime, the present approach enables direct access to Raman-active vibrational modes of ångström-scale metal-supported molecular systems. This capability is particularly significant for investigating chemically important yet IR-inactive interfacial molecules on metal surfaces, including homonuclear diatomic species such as H_2_, N_2_, and O_2_ (Table S1), whose interfacial behavior has remained challenging to probe experimentally. The pulsed excitation scheme, combined with systematic probe-delay control, also provides a foundation for time-resolved coherent Raman studies of interfacial molecular dynamics (Figure S4), including IR-inactive high-frequency vibrations.

In summary, we have developed a coherent Raman spectroscopy approach capable of sensitively detecting ångström-scale ultrathin molecular systems supported on metal surfaces without requiring plasmonic and electronic enhancement. Our method employs a time-frequency engineered CARS scheme in which femtosecond pump and Stokes pulses are followed by a time-delayed, asymmetrically shaped picosecond probe pulse, enabling significant suppression of the metal NRB. In this background-reduced regime, the residual NRB serves as an internal local oscillator, leading to coherent interferometric amplification of weak interfacial molecular vibrational signals. The directional emission of the CARS signal further enables efficient signal collection even with moderate numerical-aperture (NA) optics, facilitating practical measurements across a wide range of experimental geometries in contrast to spontaneous Raman spectroscopy, which typically requires high-NA collection optics positioned close to the sample. Overall, our approach establishes a general optical strategy for high-sensitivity interfacial Raman spectroscopy with minimal structural and environmental constraints. By complementing structure- and material-specific plasmonic and electronic enhancement strategies, it provides a versatile platform for nanoscience, surface chemistry, and molecular interface research, where ultrathin interfacial molecular layers play central functional roles.

## Supplementary Material



## Notes

Details of methods, including experimental setup, sample preparation and density functional calculation; theoretical description of time-frequency hybrid CARS spectroscopy; supporting data, including sample evaluation, damage threshold, and vibrational CARS observation (PDF)
