# Supplementary material for: Plasmonic- and Electronic-Enhancement-Free Coherent Raman Detection of Ångström-Scale Molecular Layers at Metal Interfaces
Source: Nano Lett. 2026 Apr 27;26(20):6603–10. doi: 10.1021/acs.nanolett.6c00802 (PMC13220306; doi:10.1021/acs.nanolett.6c00802)
Supplement: Supplementary file 1 [file nl6c00802_si_001.pdf]

Supporting Information

Plasmonic- and Electronic-Enhancement-Free Coherent Raman Detection of Ångström-Scale Molecular Layers at Metal Interfaces

Toshiki Sugimoto[1,2,3*], Tomoaki Ichii[1], Tsuneto Kanai[1], Ryu Yoshizawa[1,2], Shota Takahashi[1], Atsunori Sakurai[1,2,3], Keisuke Seto[1], and Chengxiang Jin [1,2]

[1]*Institute for Molecular Science, Okazaki, Aichi, 444-8585, Japan*
[2]*Graduate Institute for Advanced Studies, SOKENDAI, Okazaki, Aichi 444-8585, Japan*
[3]*Laser-driven Electron-acceleration Technology Group, RIKEN Spring-8 Center, Sayocho, Hyogo, 679-5148, Japan*
*toshiki-sugimoto@ims.ac.jp

Table of Contents

Sec. 1. Methods

Sec. 2. Preparation and Characterization of Temporally Asymmetric Picosecond Probe Pulse

Sec. 3. Time-Delay (Δt) Dependence of Non-Resonant Background Intensity: Comparison between Temporally Asymmetric and Symmetric Probe Pulses.

Sec. 4. Time-Delay (Δt) Dependence of Vibrationally Resonant Coherent Raman Intensity: Comparison between Temporally Asymmetric and Symmetric Probe Pulses.

Sec. 5. Interference between Vibrationally Non-resonant and Resonant Contributions

Sec. 6. Spectral Analysis for Extracting $\chi^{(3)}$ Vibrational Response

Sec. 7. Coherent Interferometric Amplification of the Interfacial Vibrational Signal via NRB signal from the Metal Substrate

Sec. 8. Supplementary data

Section 1: Methods

As a model system of a metal-supported ultrathin interfacial molecular layer free from plasmonic and electronic signal enhancement, a self-assembled monolayer (SAM) of benzyl mercaptan (BM) was fabricated on an atomically flat Au(111) substrate. BM-SAMs were prepared by immersing flame-annealed Au(111) in a 1 mmol L^{-1} ethanol solution of BM for 24 h. The precursor solution was visually transparent and colorless, consistent with the absence of electronic absorption in the visible region. The substrate was then rinsed with ethanol to remove weakly adsorbed or non-chemisorbed species.[1-3] Under these established preparation conditions, BM molecules chemisorb strongly onto Au through Au–S bonding and spontaneously form a self-organized, self-limited monolayer rather than multilayer aggregates or loosely bound overlayers.[1-3] Patch-like patterns characteristic of the well-defined aromatic thiolate SAMs, as typically reported in previous STM and tip-enhanced Raman studies,[1,2] were confirmed in the present study (Figure S8). These observations indicate that the resulting interface consists of a quasi-centrosymmetric phenyl ring (~5 Å thick) and an anti-centrosymmetric methylene unit (~2 Å thick), as illustrated in Figure 1(a).

All measurements were performed using near-infrared excitation, well away from the electronic resonances of both the molecular layer and the Au(111) substrate (below 540 nm). For vibrational excitation and detection, we constructed a broadband three-beam CARS system driven by a high-repetition-rate femtosecond Yb:KGW laser (1034 nm, ~190 fs, FWHM ≈ 8.5 nm, 60 μJ, 200 kHz, CARBIDE60). Three pulses were focused onto the sample with a spot diameter of approximately 50 μm using a parabolic mirror (f = 101.6 mm) at an incidence angle of 45° and spatially overlapped on the Au(111) surface: a pump pulse (ω_1, 1034 nm, fundamental, 400 nJ), a tunable broadband Stokes pulse (ω_2, 1150–1500 nm, FWHM ≈ 80 nm, 100 nJ), and a time-asymmetric narrowband probe pulse (ω_3, 1034 nm, FWHM ≈ 0.7 nm ≈ 6.3 cm^{-1}, 2.7 μJ) featuring a sharp ~280 fs rising edge and a gradual ~1.7 ps decay (Figure S1). The spectral resolution of our CARS system is dominated by the bandwidth of ω_3 (~ 6.3 cm^{-1}). The pump and Stokes pulses were incident coaxially and simultaneously, whereas the probe pulse was introduced with a tunable delay Δt (Figure 1(b)). Typical focus spot diameter of these pulses was ~80 μm.

In the reflection geometry, all incident beams and the detected anti-Stokes radiation were p-polarized. Among the possible polarization combinations, the $pppp$-CARS signal was significantly stronger than *sppp* or *sspp*, and the measured *pppp*-CARS response is dominated by the $\chi^{(3)}_{zzzz}$ component of the effective third-order non-linear susceptibility $\chi^{(3)}_{eff.pppp}$ tensor,[4,5] where z is defined as the surface-normal direction. This arises from the smaller Fresnel local field factors for in-plane (x,y) electric-field components compared with those for out-of-plane (z) components at metal interfaces.[4,5] The anti-Stokes signal was collected by a second parabolic mirror (f = 101.0 mm) in the specular reflection direction, where in-plane wavevector conservation is satisfied and the nonlinear polarization radiates constructively. The signal was directed into a Czerny–Turner

spectrometer and detected with a CCD camera. Each spectrum was acquired with an integration time of 100 s.

Because metals exhibit markedly lower laser-damage thresholds than dielectric or liquid materials, pulse energies were carefully optimized. In our configuration, irradiating the BM-SAM/Au(111) surface with the femtosecond ω_1 pulses above ~0.85 μJ (17 mJ/cm^2) resulted in sample damage (Figure S9). To avoid thermal effects or laser-induced damage while maintaining a sufficient nonlinear signal, the pulse energies of the ω_1, ω_2, and ω_3 were carefully set to 0.4 μJ (8 mJ/cm^2), 0.1 μJ (2 mJ/cm^2), and 2.7 μJ (54 mJ/cm^2), respectively. Note that the damage threshold of the ω_3 pulse was larger than that of the ω_1 pulse because of the longer pulse width.

To support the assignment of the experimentally observed vibrational features, optimized molecular structures and vibrational (IR and Raman) spectra were performed by density functional theory (DFT) calculations using the B3LYP hybrid functional and the 6-31G(d) basis set implemented in the Gaussian 16 package.[6] To account for deviations from the harmonic approximation, anharmonicity of the vibrational potential and the resulting mode mixing were explicitly treated using generalized second-order vibrational perturbation theory (GVPT2).[7,8] Transition dipole moments, transition polarizability tensors and vibrational resonance frequencies were evaluated for the anharmonically mixed vibrational modes, from which the anharmonic IR and Raman spectra shown in Figures 4, S10 and S12 were constructed. Raman activities of H_2, N_2, and O_2 molecules listed in Table S1 were calculated using the same computational protocol. Optimized molecular structures and representative vibrational modes shown in these Figures 4 and S10 were depicted using GaussView 6.[9]

Section 2: Preparation and Characterization of Temporally Asymmetric Picosecond Probe Pulse

In conventional frequency-domain nonlinear optical spectroscopy, probe pulses with durations on the order of ~1 ps or longer are typically employed to improve spectral resolution and enhance signal intensity. In the present study, we employed a time-asymmetric picosecond probe pulse, characterized by a rapid rise (several hundred femtoseconds) followed by an exponentially decaying tail lasting a few picoseconds. This uniquely shaped probe pulse was designed to effectively reduce non-resonant background (NRB) signals in coherent anti-Stokes Raman scattering (CARS) by hybridizing time- and frequency-domain spectroscopy schemes (Figures 1 and S2(a)). The ω_3 probe pulse (λ_3 = 1030 nm) was generated from the fundamental output of a Yb:KGW laser (CARBIDE, 1030 nm, 200 fs) using a Fabry–Pérot interferometer[10-13] composed of two parallel mirrors. The temporal profiles of the femtosecond fundamental pulse $E_\mathrm{f}(t)$ with Gaussian shape and the time-asymmetric picosecond probe pulse $E_3(t)$ shaped by a Fabry–Pérot interferometer are given by the following expressions:

$$E_\mathrm{f}(t) = A_\mathrm{f}\, e^{-2\ln 2(t/\Delta\tau_\mathrm{f})^2} e^{-i\omega_\mathrm{f} t}, \tag{S1}$$

$$E_3(t) = A_\mathrm{f}(1 - R_0)\sum_{n=0}^{\infty} R_0^n\; e^{-2\ln 2(t-n\tau_\mathrm{RT}/\Delta\tau_\mathrm{f})^2} e^{-i\omega_\mathrm{f}(t-n\tau_\mathrm{RT})}, \tag{S2}$$

where ω_f and $\Delta\tau_\mathrm{f}$ denote the center angular frequency and the temporal full width at the half maximum (FWHM) of the fundamental pulse, respectively. The parameters R_0 and τ_RT represent the reflectivity of the interferometer mirrors and the round-trip travel time within the Fabry–Pérot cavity, respectively, with the condition $\tau_\mathrm{RT} < \Delta\tau_\mathrm{f}$. The temporal waveform of the shaped probe pulse was experimentally evaluated by the cross correlation with a pulse of sufficiently shorter duration than $E_3(t)$. As shown in Figure S1, the experimentally evaluated temporal profile is well-simulated by Eq. (S2) with R_0=0.93 and τ_RT=124 fs, where the time axis is shifted such that the peak intensity occurs at the time origin. The spectral resolution of the probe pulse, estimated via Fourier transform of the temporal profile, is 6.3 cm^{-1}.

Note that the rise and decay portions of the time-asymmetric ω_3 probe pulse can be well characterized by a Gaussian function $\exp(-t^2/\Delta\tau_{3\mathrm{r}}^2)$ for the leading edge (t < 0) and an exponential decay function $\exp(-t/\Delta\tau_{3\mathrm{d}})$ for the trailing edge (t > 0). Accordingly, the rise and decay time profile of the electric field $E_3(t)$ is approximated by the following piecewise function:

$$E_3(t) = \begin{cases} A_0 \exp\left(-\frac{t^2}{\Delta\tau_{3r}^2}\right), & t < 0 \\ A_0 \exp\left(-\frac{t}{\Delta\tau_{3d}}\right), & t > 0 \end{cases} \tag{S3}$$

The fitting result using Eq. (S3) is shown as a dashed line in Figure S1. The extracted parameters are $\Delta\tau_{3\mathrm{r}} \approx$ 280 fs and $\Delta\tau_{3\mathrm{d}} \approx$ 1.7 ps, corresponding to a steep rise of several hundred femtoseconds followed by a picosecond-scale exponential decay. This asymmetric pulse shape allows the ω_3 probe pulse to be temporally positioned immediately after the substantial decay of

the excitation fields $E_1(t)$ and $E_2(t)$, while still maintaining sufficient temporal overlap with the vibrational coherence (Figure 1(b)). In this work, we demonstrate suppression of the strong NRB signal from metal substrate by more than four orders of magnitude, without attenuating the intrinsically weak vibrational resonant signal originating from interfacial molecular species exhibiting fast coherence decay (total dephasing time $T_2 < 1$ ps) (Figure 2, see also Supporting Information Section 2, 3 and 4).

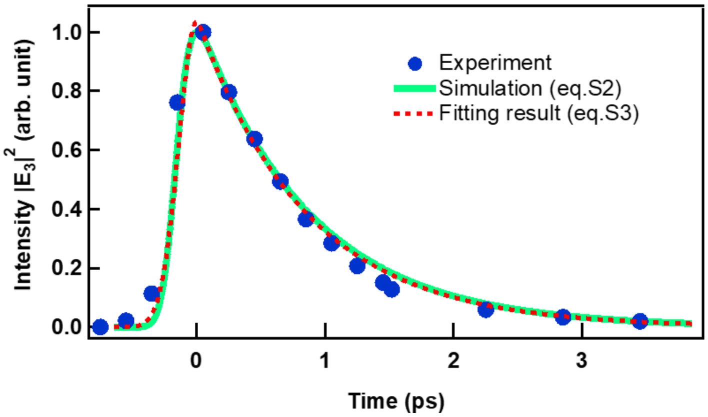

Figure S1. Comparison of the temporal profile of the experimentally evaluated ω_3 (~1030nm) probe pulse (blue circles) with the simulated waveform based on Eq. (S2) (green solid line; R_0 =0.93, τ_{RT} =124 fs) and the fitted curve using Eq. (S3) (red dashed line; $\Delta\tau_{3r} \approx$ 280 fs and $\Delta\tau_{3d} \approx$ 1.7 ps). The time origin is defined as the point at which the probe pulse intensity reaches its maximum.

Section 3: Time-Delay (Δt) Dependence of Non-Resonant Background Intensity: Comparison between Temporally Asymmetric and Symmetric Probe Pulses.

In conventional two-field coherent Raman schemes such as CARS and coherent Stokes Raman scattering (CSRS), molecular vibrations at frequency Ω_0 are coherently driven by the pump (ω_1) and Stokes (ω_2) pulses under the resonance condition $\Omega \equiv \omega_1 - \omega_2 = \Omega_0$, and the signal is generated using one of these excitation fields (ω_1 or ω_2) as the probe. Owing to their coherent nature, these techniques provide signal intensities several orders of magnitude higher than those of spontaneous Raman scattering.[14,15] However, their application to interfacial systems on metal surfaces is often hindered by strong non-resonant background (NRB) signals originating from the spatiotemporal overlap of the three optical pulses at the metal substrate within the optical penetration depth (~10 nm), which typically exceed the resonant interfacial response by several orders of magnitude. This situation can be effectively overcome by employing a three-field CARS configuration (Figure 1(b)) with a temporally asymmetric probe pulse (Figure S1).

In this section, the nonlinear signal is formulated in terms of the third-order nonlinear polarization, following the standard convention in CARS spectroscopy based on the relation $E^{(3)} \propto P^{(3)}$ and $I \propto \left|E^{(3)}\right|^2$, and the time-delay dependence of the NRB in the three-field CARS configuration is analyzed (Figure S3). Based on time dependent perturbation theory,[16,17] the vibrationally non-resonant third order nonlinear polarization generated by three incident pulses in the CARS process (Figure S2) is expressed by the vibrationally non-resonant third-order time-domain response function $R_{NR}^{(3)}(t_3\, t_2, t_1)$ as

$$P_{NR}^{(3)}(t;\tau_{12},\tau_{23}) \propto \int_0^\infty dt_3 \int_0^\infty dt_2 \int_0^\infty dt_1\, R_{NR}^{(3)}(t_3,t_2,t_1) \times E_3(t-t_3;\tau_{12}+\tau_{23}) E_2^*(t-t_3-t_2;\tau_{12}) E_1(t-t_3-t_2-t_1), \quad \text{(S4)}$$

where $E_1(t)$, $E_2(t)$, and $E_3(t)$ denote the electric fields of the three incident laser pulses. The symbol * in $E_2^*(t)$ indicates that the system interacts with the electric field proportional to $\cos(\boldsymbol{k}\cdot\boldsymbol{x} - \omega t)$ through $e^{-i(\boldsymbol{k}\cdot\boldsymbol{x}-\omega t)}$, whereas the other interactions occur through $e^{i(\boldsymbol{k}\cdot\boldsymbol{x}-\omega t)}$. As displayed in Figure S2, the integration variables t_1, t_2, and t_3 represent the intervals between the first and second pulse interactions, between the second and third pulse interactions, and between the third interaction and the detection time t, respectively, while τ_{12}, τ_{23} represent the time delays between the peaks of the pump and Stokes pulses and between those of the Stokes and probe pulses, respectively. When these interactions occur instantaneously through virtual states, the third-order response function contributing to the vibrationally non-resonant signal is given by

$$R_{NR}^{(3)}(t_3,t_2,t_1) = 2^3 \chi_{NR}^{(3)} \delta(t_3)\delta(t_2)\delta(t_1)\,, \quad \text{(S5)}$$

with $\chi_{\mathrm{NR}}^{(3)}$ being approximated as a constant.[16,17] Under this approximation, the temporal profile of the non-resonant third order nonlinear polarization simplifies to the direct product of three incident electric fields:

$$P_{NR}^{(3)}(t;\tau_{12},\tau_{23}) \propto \chi_{NR}^{(3)} E_3(t;\tau_{12}+\tau_{23}) E_2^*(t;\tau_{12}) E_1(t). \quad \text{(S6)}$$

In our experimental configuration (Figure 1(b)), the pump and Stokes pulses are incident simultaneously ($\tau_{12} = 0$), while the probe pulse is introduced with a tunable delay Δt ($\tau_{23} = \Delta t$). Under this condition, Eq. (S6) reduces to

$$P_{NR}^{(3)}(t;\Delta t) \propto \chi_{NR}^{(3)} E_3(t;\Delta t) E_2^*(t) E_1(t). \quad \text{(S7)}$$

This expression indicates that the non-resonant signal originates from the temporal overlap of the three optical pulses. In this configuration, not only the CARS scheme illustrated in Figures 1 and S2 but also the vibrationally nonresonant four-wave-mixing (FWM) process represented by $E_2^*(t)E_3(t;\Delta t)E_1(t)$ contributes significantly to the NRB signal.[16-18] For simplicity, the contribution of this process is considered to be included in Eq. (S7) in the following discussion.

From Eq. (S7), the nonresonant third-order polarization in the frequency domain, $\tilde{P}_{NR}^{(3)}(\omega,\Delta t)$, can be expressed as a double convolution of the field spectra $\tilde{E}_1$, $\tilde{E}_2^*$, and $\tilde{E}_3$, as follows:

$$\begin{aligned}
\tilde{P}_{NR}^{(3)}(\omega,\Delta t) &\propto \int_{-\infty}^{\infty} dt\, P_{NR}^{(3)}(t,\Delta t) e^{i\omega t} \\
&= \int_{-\infty}^{\infty} dt\, \chi_{NR}^{(3)} E_3(t;\Delta t) E_2^*(t) E_1(t) e^{i\omega t} \\
&\propto \chi_{NR}^{(3)} \int_{-\infty}^{\infty} dt \left[\int_{-\infty}^{\infty} d\omega_3'\, e^{-i\omega_3'(t-\Delta t)} \tilde{E}_3(\omega_3')\right] \\
&\qquad \times \left[\int_{-\infty}^{\infty} d\omega_2'\, e^{i\omega_2' t} \tilde{E}_2^*(\omega_2')\right]\left[\int_{-\infty}^{\infty} d\omega_1'\, e^{-i\omega_1' t} \tilde{E}_1(\omega_1')\right] e^{i\omega t} \\
&\propto \chi_{NR}^{(3)} \int_{-\infty}^{\infty} d\omega_3' \int_{-\infty}^{\infty} d\omega_2' \int_{-\infty}^{\infty} d\omega_1'\, \tilde{E}_3(\omega_3') \tilde{E}_2^*(\omega_2') \tilde{E}_1(\omega_1') e^{i\omega_3' \Delta t} \\
&\qquad \times \delta(\omega - \omega_3' + \omega_2' - \omega_1') \\
&\propto \chi_{NR}^{(3)} \int_{-\infty}^{\infty} d\omega_2' \int_{-\infty}^{\infty} d\omega_1'\, \tilde{E}_3(\omega - \omega_2' + \omega_1') \tilde{E}_2^*(\omega_2') \tilde{E}_1(\omega_1') e^{i(\omega-\omega_2'+\omega_1')\Delta t} \\
&\propto \chi_{NR}^{(3)} \left[\int_{-\infty}^{\infty} d\Omega\, \tilde{E}_3(\Omega+\omega) e^{i(\Omega+\omega)\Delta t}\right]\left[\int_{-\infty}^{\infty} d\omega_1'\, \tilde{E}_1(\omega_1') \tilde{E}_2^*(\omega_1' - \Omega)\right], \\
&= \chi_{NR}^{(3)} \left[\int_{-\infty}^{\infty} d\Omega\, \tilde{E}_3(\Omega+\omega) e^{i(\Omega+\omega)\Delta t}\right] C_{1,2}(\Omega),
\end{aligned} \quad \text{(S8)}$$

where $\Omega \equiv \omega_1' - \omega_2'$ and $\omega = \omega_3' - \omega_2' + \omega_1'$, $C_{1,2}(\Omega) \equiv \int_{-\infty}^{\infty} d\omega_1'\, \tilde{E}_1(\omega_1') \tilde{E}_2^*(\omega_1' - \Omega)$. For illustrative purposes, it is instructive to consider the limiting case in which the $\tilde{E}_3$ has a much narrower bandwidth than $\tilde{E}_1$ and $\tilde{E}_2^*$ and can be approximated by a delta function centered at ω_3 as $\tilde{E}_3(\omega) \approx \tilde{E}_{3,0}\delta(\omega - \omega_3)$. Under this condition, the $\tilde{P}_{NR}^{(3)}$ spectroscopic response reduces to a simple convolution of the $\tilde{E}_1$ and $\tilde{E}_2^*$ spectra,

$$\tilde{P}_{NR}^{(3)}(\omega;\Delta t) \propto \chi_{NR}^{(3)}\tilde{E}_{3,0}\left[\int_{-\infty}^{\infty} d\Omega\, \delta(\Omega+\omega-\omega_3)e^{i(\Omega+\omega)\Delta t}\right]C_{1,2}(\Omega)$$

$$= \chi_{NR}^{(3)}\tilde{E}_{3,0}e^{i\omega_3\Delta t}C_{1,2}(\Omega), \tag{S9}$$

where, $\omega = \omega_3 - \Omega$. This simplified expression shows that the spectral profile of the NRB is mainly determined by the convolution of the spectra of pump and Stokes pulses $C_{1,2}(\Omega) = \int_{-\infty}^{\infty} d\omega_1'\, \tilde{E}_1(\omega_1')\tilde{E}_2^*(\omega_1'-\Omega)$ (Figure 2(a)).

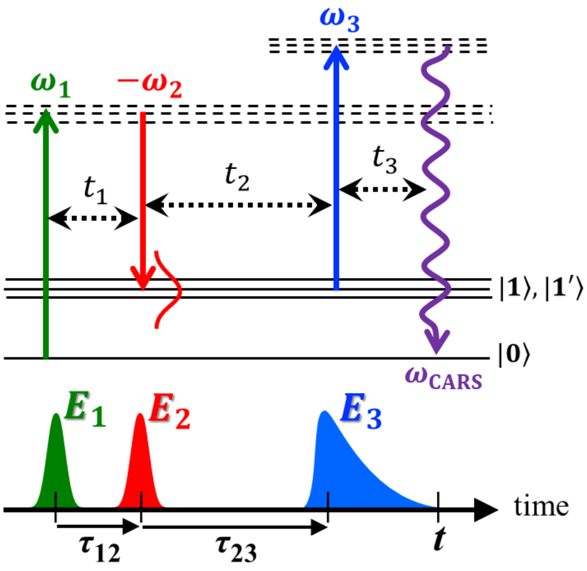

Figure S2. Wave-mixing energy-level diagram depicting the third-order response function, $R^{(3)}(t_3, t_2, t_1)$ that dominates CARS observations. Solid vertical arrows represent field interactions with pulses E_1(pump), E_2(Stokes), and E_3(probe), whereas the wavy arrow represents the emitted CARS field. The integration variables t_1, t_2, and t_3 reflect the coherence timescales separating light-matter interactions. τ_{12}, τ_{23} represent the time delays between the pump and Stokes pulses and between the Stokes and probe pulses, respectively. In our experimental configuration (Figure 1(b)), the pump and Stokes pulses are incident simultaneously ($\tau_{12} = 0$), while the probe pulse is introduced with a tunable delay Δt ($\tau_{23} = \Delta t$).

We first consider an experimentally relevant condition in which the pump $E_1(t)$ and Stokes $E_2(t)$ fields are approximated as Gaussian temporal envelopes with temporal full widths at half maximum (FWHM) $\Delta\tau_1$ and $\Delta\tau_2$, respectively, while the probe field $E_3(t;\Delta t)$ has an asymmetric temporal profile (Figure S1):

$$E_1(t) = A_1 e^{-2ln2\left(\frac{t}{\Delta\tau_1}\right)^2} e^{-i\omega_1 t}\ , \tag{S10}$$

$$E_2(t) = A_2 e^{-2ln2\left(\frac{t}{\Delta\tau_2}\right)^2} e^{-i\omega_2 t}, \tag{S11}$$

$$E_3(t;\Delta t) = A_{\mathrm{f}}(1-R_0)\sum_{n=0}^{\infty} R_0^n\ e^{-2\ln 2\left((t-\Delta t)-n\tau_{\mathrm{RT}}/\Delta\tau_{\mathrm{f}}\right)^2} e^{-i\omega_{\mathrm{f}}\left((t-\Delta t)-n\tau_{\mathrm{RT}}\right)}. \tag{S12}$$

When the pump and Stokes pulse durations $\Delta\tau_1$ and $\Delta\tau_2$ are on the order of a few femtoseconds and are comparable to the steep rise time of the probe pulse $E_3(t;\Delta t)$, and when the frequency condition ω_1=ω_3=ω_f is satisfied, the third-order nonlinear polarization contributing to the NRB signal is derived from Eq. (S7) as

$$P_{\mathrm{NR}}^{(3)}(t;\Delta t) \propto \chi_{NR}^{(3)} e^{-i(\omega_1-\omega_2+\omega_3)t} e^{-2\ln2\left(\frac{t}{\Delta\tau_1}\right)^2 - 2\ln2\left(\frac{t}{\Delta\tau_2}\right)^2}$$

$$\times (1-R_0)\sum_{n=0}^{\infty} R_0^n e^{-2\ln2\left(\frac{t-\Delta t-n\tau_{\mathrm{RT}}}{\Delta\tau_f}\right)^2} e^{i\omega_3(\Delta t+n\tau_{\mathrm{RT}})}. \quad \text{(S13)}$$

When the temporal widths $\Delta\tau_1$, $\Delta\tau_2$, $\Delta\tau_{\mathrm{f}}$ are approximately equal ($\Delta\tau_1 = \Delta\tau_2 = \Delta\tau_{\mathrm{f}} \equiv \Delta\tau$), the third-order nonlinear polarization contributing to the NRB signal can be simplified as

$$P_{\mathrm{NR}}^{(3)}(t;\Delta t) \propto e^{-i(\omega_1-\omega_2+\omega_3)t} \times (1-R_0)\sum_{n=0}^{\infty} R_0^n e^{-4\ln2\left(\frac{t}{\Delta\tau}\right)^2} e^{-2\ln2\left(\frac{t-\Delta t-n\tau_{\mathrm{RT}}}{\Delta\tau}\right)^2} e^{i\omega_3(\Delta t+n\tau_{\mathrm{RT}})}. \quad \text{(S14)}$$

The corresponding NRB intensity is given by

$$I_{\mathrm{NRB}}^{(3)}(\omega;\Delta t) \propto \left|\tilde{P}_{\mathrm{NR}}^{(3)}(\omega;\Delta t)\right|^2 = \left|\int_{-\infty}^{\infty} dt e^{i\omega t} P_{\mathrm{NR}}^{(3)}(t;\Delta t)\right|^2$$

$$\propto \left|(1-R_0)\sum_{n=0}^{\infty} R_0^n e^{-\frac{4\ln2}{3}\left(\frac{\Delta t+n\tau_{\mathrm{RT}}}{\Delta\tau}\right)^2} e^{i\omega_f(\Delta t+n\tau_{\mathrm{RT}})}\right|^2. \quad \text{(S15)}$$

A numerical calculation result for the temporal widths of $\Delta\tau$ = 200 fs is shown in Figure 2(b) (solid curve) and Figure S3(c) (red solid curve). Notably, the NRB intensity decreases by more than four orders of magnitude at a probe delay $\Delta t \sim 3\Delta\tau$ = 600 fs, in excellent agreement with experimental result. In this delay region, although the main pulse peaks are largely separated, the residual partial overlap of the low-intensity pulse tails give rise to the much weakened but finite NRB signal.

Although the use of such time-asymmetric picosecond probe pulse (Figures S1 and S3(a)) enables strong NRB signal suppression at sub-picosecond delays, such effective suppression at sub-picosecond Δt region cannot be achieved using a conventional time-symmetric picosecond probe pulse[19-21] (Figure S3(b)). To quantitatively compare these two cases, we consider a typical Gaussian-shaped probe pulse $E_3(t;\Delta t) = A_3 e^{-2\ln2((t-\Delta t)/\Delta\tau_3)^2} e^{-i\omega_3 t}$ with temporal withdth $\Delta\tau_3$=2.3 ps, yielding a frequency resolution of 6.3 cm^{-1} comparable to that of the time-asymmetric prove pulse shown in Figure S1. In this case, the NRB polarization and intensity are given as,

$$P_{\mathrm{NR}}^{(3)}(t;\Delta t) \propto e^{-i(\omega_1-\omega_2+\omega_1)t} e^{-4\ln2(t/\Delta\tau)^2 - 2\ln2[(t-\Delta t)/\Delta\tau_3]^2}, \quad \text{(S16)}$$

$$I_{\mathrm{NRB}}^{(3)}(\omega;\Delta t) \propto \left|\tilde{P}_{\mathrm{NR}}^{(3)}(\omega;\Delta t)\right|^2$$

$$= \left|\int_{-\infty}^{\infty} dt e^{i\omega t} P_{\mathrm{NR}}^{(3)}(t;\Delta t)\right|^2$$

$$\propto \left|e^{b(\Delta t)^2/a - c(\Delta t)}\right|^2, \quad \text{(S17)}$$

where $a = 2/\Delta\tau^2 + 1/\Delta{\tau_3}^2$, $b(\Delta t) = \sqrt{2\ln2}\,\Delta t/\Delta{\tau_3}^2$, $c(\tau_3) = 2\ln2\Delta t^2/\Delta{\tau_3}^2$. The calculated Δt delay-time dependence of the NRB intensity is shown in Figure S3(c) (green solid line). The result indicates that, with the commonly used time-symmetric picosecond probe pulse (Figure S3(b)), achieving effective NRB suppression of more than three orders of magnitude

requires temporal delays of several picoseconds or longer, which significantly exceeds the fast vibrational relaxation time (total dephasing time $T_2 < 1$ ps) of typical interfacial molecular modes on metal surfaces. Therefore, with such a probe pulse, it becomes fundamentally difficult to observe the intrinsically weak resonant CARS signals from rapidly dephasing interfacial molecular vibrations (see also next section).

It is worth noting that the CSRS process shows essentially the same Δt dependence of the NRB intensity as the CARS process, reflecting their analogous third-order nonlinear interaction pathways.[22] Therefore, the present analysis and conclusions are generally applicable to both CARS and CSRS schemes.

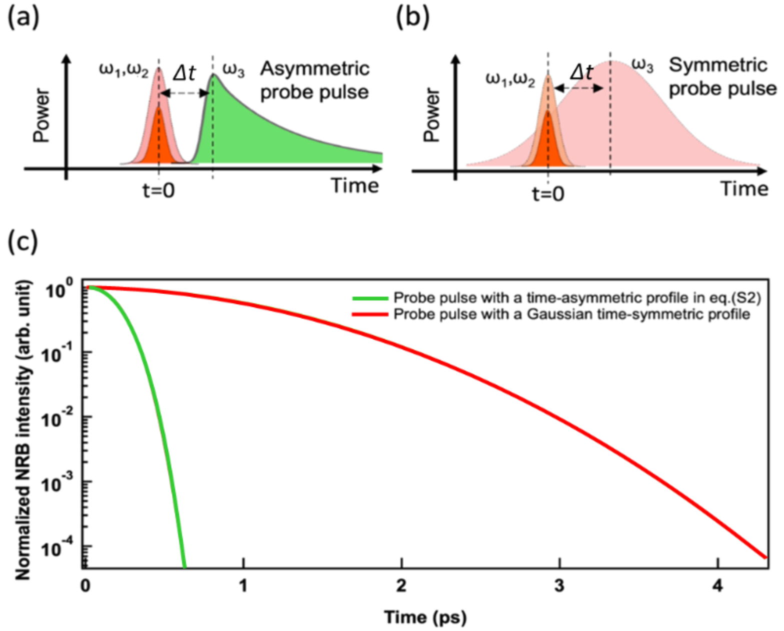

Figure S3. (a,b) Three-field CARS scheme using a probe pulse with a time-asymmetric picosecond width (a) and with a time-symmetric picosecond width (b). (c) Comparison of the time-delay Δt dependence of the decay profile of NRB intensity calculated by the time-asymmetric picosecond pulse (Eq. S15, red solid curve) and time-symmetric picosecond Gaussian pulse with temporal width $\Delta\tau_3$=2.3 ps (Eq. S17, green solid curve).

Section 4: Time-Delay (Δt) Dependence of Vibrationally Resonant Coherent Raman Intensity: Comparison between Temporally Asymmetric and Symmetric Probe Pulses.

Similar to the case of vibrationally non-resonant condition (Section 3), the third-order nonlinear polarization contributing to vibrationally resonant CARS signal is expressed as,

$$P_R^{(3)}(t;\tau_{12},\tau_{23}) \propto \int_0^\infty dt_3 \int_0^\infty dt_2 \int_0^\infty dt_1 R_R^{(3)}(t_3,t_2,t_1) \times E_3(t-t_3;\tau_{12}+\tau_{23})E_2^*(t-t_3-t_2;\tau_{12})E_1(t-t_3-t_2-t_1), \quad \text{(S18)}$$

where $R_R^{(3)}(t_3,t_2,t_1)$ is the vibrationally resonant third-order time-domain response function and the integration variables t_1, t_2, and t_3 denote time intervales between pulse interactions (Figure S2). We focus on the case where the pump (ω_1) and Stokes (ω_2) pulses induce vibrational coherence characterized by a center frequency of Ω_0 and a total dephasing time T_2, and the ω_3 probe pulse converts this coherence into the anti-Stokes Raman signal. In particular, we consider the situation where the pump and probe pulses are largely detuned from electronic resonance (Figures 1(b) and S2), so that their interactions occur instantaneously through virtual states on both the t_1 and t_3 time scales.[16,17] In this case, $R_R^{(3)}(t_3,t_2,t_1)$ is approximated as

$$R_R^{(3)}(t_3,t_2,t_1) = \delta(t_3)R_R^{(3)}(0,t_2,0)\delta(t_1), \quad \text{(S19)}$$

$$R_R^{(3)}(0,t_2,0) \propto i\theta(t_2)\exp(-i\Omega_0 t_2 - t_2/T_2), \quad \text{(S20)}$$

where $\theta(t_2)$ is the Heaviside step function.[16,17] Then, Eq. (S18) is transformed as

$$P_R^{(3)}(t;\tau_{12},\tau_{23}) \propto E_3(t;\tau_{12}+\tau_{23}) \int_0^\infty dt_2 R_R^{(3)}(0,t_2,0)E_2^*(t-t_2;\tau_{12})E_1(t-t_2). \quad \text{(S21)}$$

In our experimental configuration (Figure 1(b)), the pump and Stokes pulses are incident simultaneously ($\tau_{12}=0$), while the probe pulse is introduced with a tunable delay Δt ($\tau_{23}=\Delta t$). Under this condition, Eq. (S21) reduces to

$$P_R^{(3)}(t;\Delta t) \propto E_3(t;\Delta t) \int_0^\infty dt_2 R_R^{(3)}(0,t_2,0)E_2^*(t-t_2)E_1(t-t_2). \quad \text{(S22)}$$

In the present case, the temporal width of the pump and Stokes pulses are much shorter than T_2. Therefore, to clarify the essential temporal relationship between the molecular response and the probe field, here we formally replace their field envelopes with delta functions and derive a simplified analytical form. Under this condition, the time-domain resonant third-order nonlinear polarization is expressed as

$$P_R^{(3)}(t;\Delta t) \propto E_3(t;\Delta t)R_R^{(3)}(0,t,0). \quad \text{(S23)}$$

In this framework, the delayed probe (up-conversion) pulse effectively serves as a variable temporal gate that samples the temporally evolving molecular vibrational response (Figure S4(a)).

When the leading-edge temporal width $\Delta\tau_{3r}$ of the time-asymmetric probe field (Figures S1, Eq. (S3)) is also substantially shorter than T_2, the frequency-domain representation of $\tilde{P}_R^{(3)}(\omega;\Delta t)$ can be approximated as in Eq. (S25) through Eq. (S24), as described below.

$$\begin{aligned}
\tilde{P}_R^{(3)}(\omega;\Delta t) &\propto \mathcal{F}\left\{P_R^{(3)}(t;\Delta t)\right\} = \mathcal{F}\left\{E_3(t;\Delta t)R_R^{(3)}(0,t,0)\right\} \\
&= \mathcal{F}\left\{E_3(t)R_R^{(3)}(0,t+\Delta t,0)\right\} \\
&\propto \exp(-\Delta t/T_2)\mathcal{F}\left\{E_3(t)R_R^{(3)}(0,t,0)\right\} \\
&\propto \exp(-\Delta t/T_2)\int_{-\infty}^{\infty} dt\, e^{i\omega t}E_3(t)R_R^{(3)}(0,t,0) \\
&\propto \exp(-\Delta t/T_2)\int_{-\infty}^{\infty} dt\, e^{i\omega t}\{\int_{-\infty}^{\infty} d\omega'\, e^{-i\omega' t}\tilde{E}_3(\omega')\}R_R^{(3)}(0,t,0) \\
&= \exp(-\Delta t/T_2)\int_{-\infty}^{\infty} d\omega'\,\tilde{E}_3(\omega')\chi_R^{(3)}(\omega-\omega'),
\end{aligned} \tag{S24}$$

where $\chi_R^{(3)}(\Omega) \equiv \int_{-\infty}^{\infty} d\,t e^{i\Omega t}R_R^{(3)}(0,t,0) = A_0/(\Omega_0 - \Omega - i/T_2)$ is the vibrationally resonant third-order nonlinear susceptibility. When the temporal width of the probe pulse is sufficiently longer than T_2, the probe bandwidth becomes narrower than the linewidth of the vibrational mode. Under this condition, for mathematical simplification, the spectral profile of the probe field can be approximated as a delta function $E_3(\omega') \propto \delta(\omega' - \omega_3)$ and $\tilde{P}_R^{(3)}(\omega,\tau_3)$ can be further simplified as:

$$\tilde{P}_R^{(3)}(\omega;\Delta t) \propto \exp(-\Delta t/T_2)\chi_R^{(3)}(\omega-\omega_3). \tag{S25}$$

Therefore, the third-order nonlinear polarization and the vibrationally resonant CARS intensity spectrum at $\omega = \omega_{\mathrm{CARS}} \equiv \omega_3 + \Omega$ exhibits the following Δt delay-time dependence as a function of the Raman shift $\Omega \equiv \omega_1 - \omega_2$:

$$\tilde{P}_R^{(3)}(\omega_{\mathrm{CARS}};\Delta t) \propto \exp(-\Delta t/T_2)\chi_R^{(3)}(\Omega), \tag{S26}$$

$$I_R^{(3)}(\omega_{\mathrm{CARS}};\Delta t) \propto \left|\tilde{P}_R^{(3)}(\omega_{\mathrm{CARS}};\Delta t)\right|^2 \propto \exp(-2\Delta t/T_2)\left|\chi_R^{(3)}(\Omega)\right|^2. \tag{S27}$$

The exponential decay behavior is calculated in Figures S4(c) and S5(d) with a representative total dephasing time of T_2 =0.7 ps. While the vibrationally resonant CARS signal exhibits only a modest reduction by a factor of 2–3 at a sub-picosecond delay (e.g., Δt ~ 500 fs), it is significantly attenuated by 2–3 orders of magnitude at probe delays of several picoseconds. This highlights the critical importance of employing probe pulses that allow access to the short-delay regime while drastically reducing non-resonant signals, for the efficient detection of rapidly dephasing interfacial vibrational modes.

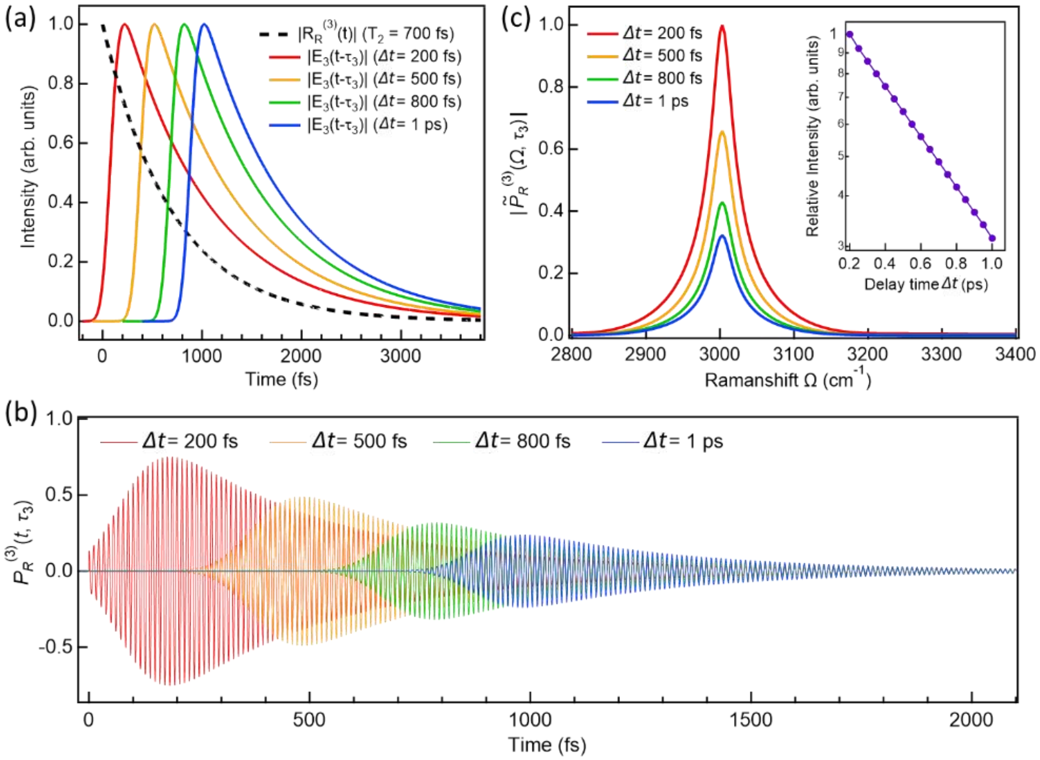

Figure S4. Δt-dependent time- and frequency-domain profiles of the vibrationally resonant CARS signal. (a) Time-domain electric field envelopes of the time-delayed probe pulse, $E_3(t;\Delta t)$, shown in Figure S1, and the vibrational response function $\left|R_R^{(3)}(0,t,0)\right| \propto \theta(t)\exp(-t/T_2)$ with T_2= 0.7 ps. (b) Simulated time-domain resonant polarization, $P_R^{(3)}(t;\Delta t)$, numerically calculated from Eq. (S23) using the time-delayed probe field $E_3(t;\Delta t)$ and the vibrational response function $R_R^{(3)}(0,t,0)$ with Ω_0 = 3000 cm^{-1} and T_2 = 0.7 ps. (c) Corresponding frequency-domain $\left|\tilde{P}_R^{(3)}(\Omega;\Delta t)\right|$ CARS spectra at various Δt. (inset) τ_3 dependence of the peak intensity of $\left|\tilde{P}_R^{(3)}(\Omega;\Delta t)\right|$. The slope of the semilogarithmic plot is consistent with the total dephasing time of T_2= 0.7 ps.

In particular, this approach uniquely hybridizes frequency-domain spectral acquisition with time-domain control.[16,17] Specifically, the use of a narrowband picosecond probe with a steep temporal rise (Figure S1) allows for direct acquisition of well-resolved vibrational spectra similar to conventional frequency-domain CARS, while the tunable probe delay introduces a time-domain window that selectively suppresses the prompt NRB, a benefit typically associated with femtosecond time-resolved spectroscopy. In this way, the time-delayed three-beam CARS scheme integrates the spectral detection in frequency domain with the temporal gating capability of time-domain techniques, enabling background-controlled and high-fidelity detection of fast-dephasing interfacial vibrational modes (Figure 2-4). Furthermore, by carefully tuning the probe delay to leave a residual overlap of the three pulses, it becomes possible to retain a controlled amount of

non-resonant background, which then acts as an internal local oscillator.[10,23,24] This configuration allows the vibrationally resonant signal to undergo interference with the internally generated non-resonant field, thereby amplifying the resonant CARS signal with phase sensitivity (see next section).

In contrast, in conventional two-beam CARS schemes where the pump and probe fields are typically derived from the same narrowband picosecond or nanosecond laser source, inherently lack the ability to temporally decouple the prompt NRB response from the resonant vibrational response. As a result, this approach severely limits NRB suppression without simultaneously attenuating the vibrational signals.

It is also worth noting that almost the same Δt delay-time dependence of the NRB and vibrationally resonant signals in CSRS,[22] with a slight difference in the vibrationally resonant susceptibility expression: $\chi_R^{(3)}(\Omega) = A_0/(\Omega_0 - \Omega + i/T_2)$.

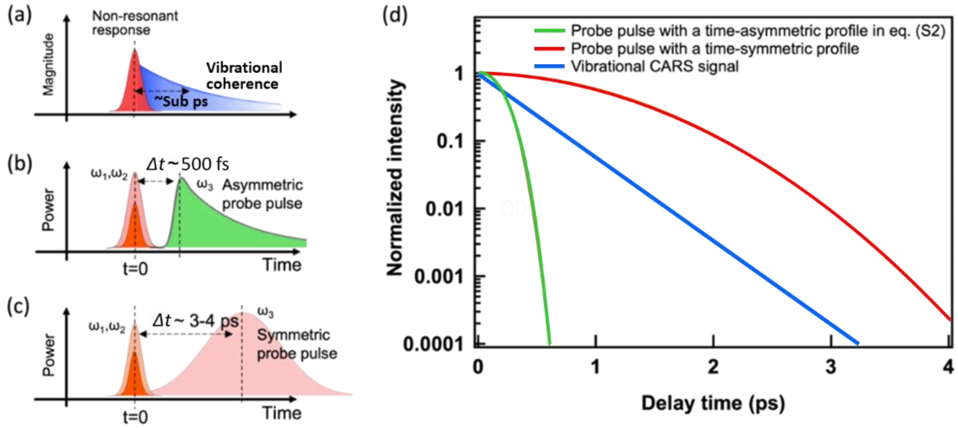

Figure S5. (a) Schematic illustration of the temporal response of a vibrational coherence (blue) and nonresonant background (red). (b and c) Three-beam CARS scheme with a symmetric Gaussian probe pulse with a picosecond temporal width (b), and a time-asymmetric probe pulse shown in Figure S1 (c). (d) Calculated Δt time-delay dependence of the vibrational resonant CARS intensity using Eq. (S27, blue line) with a typical dephasing time of $T_2 = 0.7$ ps. Also shown are the NRB decay profiles calculated using the time-asymmetric probe pulse (Eq. S15, red curve) and the symmetric Gaussian probe pulse with temporal width $\Delta\tau_3$=2.3 ps (Eq. S17, green curve), as presented in Figure S3.

Section 5: Interference between Vibrationally Non-resonant and Resonant Contributions

The experimentally detected CARS signal arises from the interference between the radiated third-order fields. The Raman-shift $\Omega \equiv \omega_1 - \omega_2$ dependence of the frequency-domain third-order CARS signal at $\omega = \omega_{\mathrm{CARS}} \equiv \omega_3 + \Omega$ and at a probe delay time Δt is expressed by the coherent sum of the vibrationally nonresonant and resonant components:

$$\tilde{E}^{(3)}_{\mathrm{CARS}}(\Omega;\Delta t) = \tilde{E}^{(3)}_{\mathrm{NR}}(\Omega;\Delta t) + \tilde{E}^{(3)}_{\mathrm{R}}(\Omega;\Delta t), \tag{S28}$$

where $\tilde{E}^{(3)}_{\mathrm{NR}}$ and $\tilde{E}^{(3)}_{\mathrm{R}}$ are proportional to the corresponding third-order polarizations $\tilde{P}^{(3)}_{\mathrm{NR}}$ and $\tilde{P}^{(3)}_{\mathrm{R}}$, respectively. The frequency-domain polarizations are given via Fourier transformation as $\tilde{P}^{(3)}_{\mathrm{NR}}(\Omega;\Delta t) = \int_{-\infty}^{\infty} \mathrm{d}t e^{i\Omega t} P^{(3)}_{\mathrm{NR}}(t;\Delta t)$, $\tilde{P}^{(3)}_{\mathrm{R}}(\Omega;\Delta t) = \int_{-\infty}^{\infty} \mathrm{d}t e^{i\Omega t} P^{(3)}_{\mathrm{R}}(t;\Delta t)$. When the probe delay Δt is shorter than the temporal rise time of the probe pulse $\Delta\tau_{r3}$ (Eq. (S3), Figure S1), the CARS signal is dominated by the NRB (Figures. 2(a)), which arises from the temporal overlap of the three input pulses (Eq. (S7)). In contrast, at delays $\Delta t \sim 2\Delta\tau_{r3} = 560$ fs, the metal NRB is substantially suppressed (Figure S3) and typically becomes only an order of magnitude larger than the resonant signal. Under this condition, interference between the vibrationally resonant and nonresonant components clearly appears (inset of Figure 2(a)).

This interference effect is experimentally observed in the CARS spectra: at a probe delay of Δt~500 fs, the vibrational resonance associated with the C-H stretching mode of the phenyl ring near ~3070 cm^{-1} exhibits a pronounced dispersive (derivative-like) lineshape (inset of Figure 2(a), Figure 3(a)). Under the condition $\left|P^{(3)}_{NR}\right| \gg \left|P^{(3)}_{\mathrm{R}}\right|$, the CARS intensity in the frequency domain at τ_3 can be approximated as

$$\begin{aligned} I_{\mathrm{CARS}}(\Omega;\Delta t) &\propto \left|\tilde{E}^{(3)}_{\mathrm{CARS}}(\Omega;\Delta t)\right|^2 \propto \left|\tilde{P}^{(3)}_{\mathrm{CARS}}(\Omega;\Delta t)\right|^2 = \left|\tilde{P}^{(3)}_{\mathrm{NR}}(\Omega;\Delta t) + \tilde{P}^{(3)}_{\mathrm{R}}(\Omega;\Delta t)\right|^2 \\ &\approx \left|\tilde{P}^{(3)}_{\mathrm{NR}}(\Omega;\Delta t)\right|^2 + 2\mathrm{Re}\left[\tilde{P}^{(3)*}_{\mathrm{NR}}(\Omega;\Delta t)\tilde{P}^{(3)}_{\mathrm{R}}(\Omega;\Delta t)\right]. \end{aligned} \tag{S29}$$

By defining ϕ_{NR} as the phase of the $\tilde{P}^{(3)}_{\mathrm{NR}}(\Omega;\Delta t)$, Eq. (S29) can be further written as

$$I_{\mathrm{CARS}}(\Omega;\Delta t) \approx \left|\tilde{P}^{(3)}_{\mathrm{NR}}(\Omega;\Delta t)\right|^2 + 2\left|\tilde{P}^{(3)}_{\mathrm{NR}}(\Omega;\Delta t)\right|\mathrm{Re}\left[e^{-i\phi_{NR}}\tilde{P}^{(3)}_{\mathrm{R}}(\Omega;\Delta t)\right]. \tag{S30}$$

The second term of Eq. (S30) shows that the non-resonant polarization acts as an internal local oscillator, enabling coherent interferometric amplification of the weak vibrationally resonant signal.[10,23,24] However, this interference gives rise to the complex spectral lineshape, such as dispersive feature, in the observed CARS spectrum (inset of Figure 2(a)). To isolate and visualize this effect more clearly, we normalize the total CARS intensity spectrum $I_{\mathrm{CARS}}(\Omega;\Delta t)$ by the pure

. (S31)

NRB intensity spectrum $I_{\mathrm{NRB}}(\Omega, \tau_3) \propto \left|P_{\mathrm{NR}}^{(3)}(\Omega; \Delta t)\right|^2$, yielding the following approximate expression:

$$\frac{I_{\mathrm{CARS}}(\Omega; \Delta t)}{I_{\mathrm{NRB}}(\Omega; \Delta t)} \approx 1 + \frac{2}{\left|\tilde{P}_{\mathrm{NR}}^{(3)}\right|} \mathrm{Re}\left[e^{-i\phi_{NR}} \tilde{P}_{\mathrm{R}}^{(3)}(\Omega; \Delta t)\right]$$

Nore that when the leading-edge temporal width $\Delta\tau_{3\mathrm{r}}$ of the time-asymmetric probe field (Eq. (S3) and Figure S1) is much shorter than the total dephasing time T_2, and the spectral bandwidth of the probe pulse is narrower than the linewidth of the vibrational mode, as assumed in Eq. (S26), a simple expression is given relating the resonant third-order polarization to the third-order vibrationally resonant susceptibility as $\tilde{P}_R^{(3)}(\Omega; \Delta t) = P_{R0}^{(3)} \exp(-\Delta t/T_2) \chi_R^{(3)}(\Omega)$, where $\chi_R^{(3)}(\Omega) = A_0/(\Omega_0 - \Omega - i/T_2)$. Under this condition, the normalized CARS intensity spectrum becomes

$$\frac{I_{\mathrm{CARS}}(\Omega; \Delta t)}{I_{\mathrm{NRB}}(\Omega; \Delta t)} \approx 1 + \frac{2P_{R0}^{(3)} \exp(-\Delta t/T_2)}{\left|P_{\mathrm{NR}}^{(3)}\right|} \mathrm{Re}\left[e^{-i\phi_{NR}} \chi_R^{(3)}(\Omega)\right]$$

$$= 1 + S(\Delta t) \mathrm{Re}\left[e^{-i\phi_{NR}} \chi_R^{(3)}(\Omega)\right], \quad \text{(S32)}$$

where $S(\Delta t) \equiv 2P_{R0}^{(3)} \exp(-\Delta t/T_2)/\left|P_{\mathrm{NR}}^{(3)}\right|$. The spectral shape of the normalized CARS intensity is thus governed by $\mathrm{Re}\left[e^{-i\phi_{NR}} \chi_R^{(3)}(\Omega)\right]$. When $\phi_{NR} = 0$ or π, vibrational response of the normalized CARS intensity follows $\mathrm{Re}\chi_R^{(3)}(\Omega)$, resulting in a dispersive (derivative-like) line shape (Figure S6(a)). In contrast, when $\phi_{NR} = \pm\pi/2$, the normalized CARS spectrum reflects $\mathrm{Im}\chi_R^{(3)}(\Omega)$, leading to purely absorptive peak (Figure S6(b)).

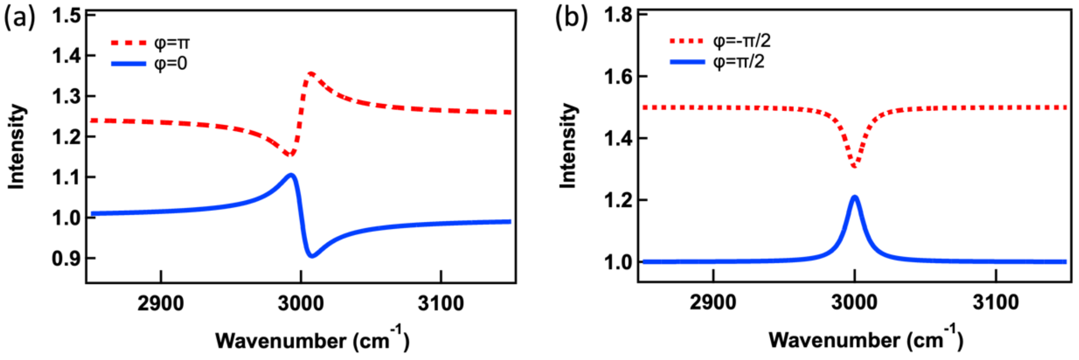

Figure S6. Simulated normalized CARS intensity spectra using Eq. (S32) with parameters Ω_0= 3000 cm^{-1}, Γ_0= 15 cm^{-1} for (a) $\phi_{NR} = 0$ or π, and (b) $\phi_{NR} = \pm\pi/2$. The spectra are vertically offset for visual clarity.

Section 6: Spectral Analysis for Extracting $\chi^{(3)}$ Vibrational Response

To extract the vibrational contribution from the total CARS signal, we also measured the pure NRB intensity spectrum $I_{\mathrm{NRB}}(\Omega;\Delta t)$ by performing time-delayed three-beam CARS measurement on a bare Au(111) substrate under the same measurement conditions (inset of Figure 2(a)). Then, the vibrationally resonant component (Figure 3(b)) was extracted by analyzing the normalized CARS intensity spectrum $I_{\mathrm{CARS}}(\Omega;\Delta t)/I_{\mathrm{NRB}}(\Omega;\Delta t)$ (Eq. (S32)) shown in Figure 3(a), using the maximum-entropy method.[25,26]

Subtracting unity from the normalized CARS intensity spectrum (Figure S7(a)), i.e., obtaining $(I_{\mathrm{CARS}}/I_{\mathrm{NRB}})-1$, which corresponds to normalization of the difference CARS intensity spectrum by $I_{\mathrm{NRB}}(\Omega;\Delta t)$, gives the simple form of vibrationally resonant third order susceptibility $\chi_R^{(3)}$ as,

$$\frac{I_{\mathrm{CARS}}(\Omega;\Delta t)}{I_{\mathrm{NRB}}(\Omega;\Delta t)}-1=\frac{I_{\mathrm{CARS}}(\Omega;\Delta t)-I_{\mathrm{NRB}}(\Omega;\Delta t)}{I_{\mathrm{NRB}}(\Omega;\Delta t)}=S(\Delta t)\mathrm{Re}\left[e^{-i\phi_{NR}}\chi_R^{(3)}(\Omega)\right]. \quad \text{(S33)}$$

This normalized difference CARS spectrum reflects the real (dispersive) or imaginary (absorptive) part of $\chi_R^{(3)}$, depending on the phase ϕ_{NR}. Therefore, to obtain $\chi_R^{(3)}$, a simple alternative approach is to fit the normalized differential spectrum (Figure S7(a)) using the Lorentzian sum model, $\chi_R^{(3)}(\Omega)=\sum_k A_{0k}/(\Omega_{0k}-\Omega-i/T_{2k})$, assuming multiple interfacial vibrational modes. The resulting $\mathrm{Re}\chi_R^{(3)}(\Omega)$ and $\mathrm{Im}\chi_R^{(3)}(\Omega)$ spectra (Figure S7(c)) show excellent agreement with those obtained from the maximum-entropy analysis[25,26] of the normalized CARS intensity spectrum without assuming any predefined lineshape (Figures 3(b), S7(b)). Importantly, the $\mathrm{Im}\chi_R^{(3)}(\Omega)$ spectrum exhibits an absorptive vibrational resonant profile that directly corresponds to a conventional spontaneous Raman spectrum.[25] Therefore, we have succeeded in obtaining the Raman spectra of Ångström-scale ultrathin interfacial molecular systems on atomically flat metal surfaces, without relying on electronic and plasmonic enhancement effects (Figures 3(b), 4, S10, and S11).

For comparison, note that in CSRS, vibrationally resonant susceptibility is expressed by $\chi_R^{(3)}(\Omega)=\sum_k A_{0k}/(\Omega_{0k}-\Omega+i/T_{2k})$, indicating that the sign of the imaginary part is opposite to that in CARS.[22]

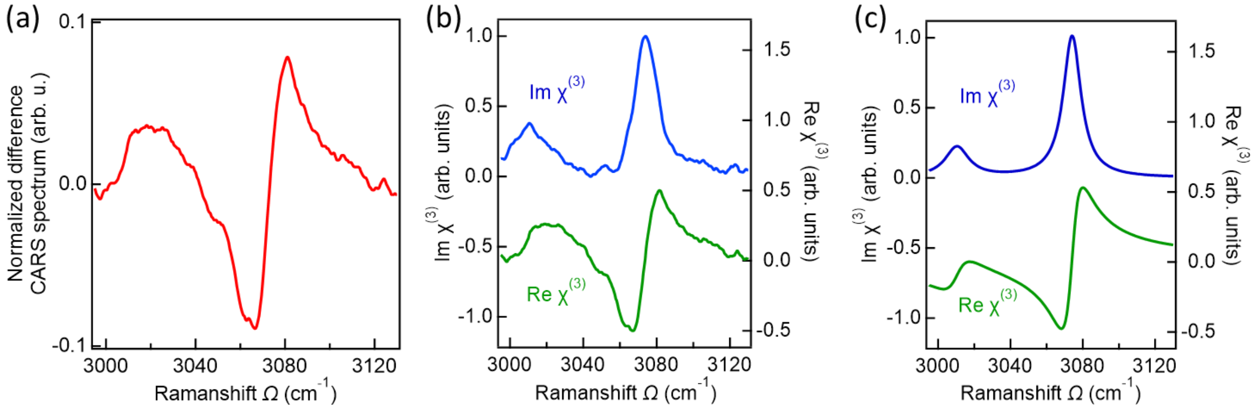

Figure S7. (a) Normalized difference CARS intensity spectrum, $(I_{\mathrm{CARS}}/I_{\mathrm{NRB}})-1$, obtained at a probe delay of Δt= 500 fs. (b) The Re$\chi_R^{(3)}(\Omega)$ and Im$\chi_R^{(3)}(\Omega)$ spectra retrieved from the maximum entropy analysis[25,26] of the normalized difference CARS intensity spectrum shown in (a). (c) For comparison, the Re$\chi_R^{(3)}(\Omega)$ and Im$\chi_R^{(3)}(\Omega)$ spectra extracted from a fitting analysis using two Lorentzian components, $\chi_R^{(3)}(\Omega)=\sum_{k=1,2}A_{0k}/(\Omega_{0k}-\Omega-i/T_{2k})$, with a nonresonant phase of $\phi_{NR}\approx\pi$.

Section 7: Coherent Interferometric Amplification of the Interfacial Vibrational Signal via NRB signal from the Metal Substrate

As discussed in section S5, interference between the vibrationally resonant and nonresonant components can serve as a coherent amplification of the vibrational signal. Note that the NRB is not intentionally introduced as a separate local oscillator in the present study, but corresponds to the non-resonant response from the metal substrate that naturally remains after substantial suppression by the probe-delay scheme (Figures 2 and S3). When the NRB signal is substantially stronger than the vibrationally resonant contribution, the CARS signal at a given probe delay time Δt is expressed from Eq. (S29) as

$$I_{\mathrm{CARS}}(\Omega;\Delta t)\approx\left|\tilde{P}_{\mathrm{NR}}^{(3)}(\Omega;\Delta t)\right|^2+2\left|\tilde{P}_{\mathrm{NR}}^{(3)}(\Omega;\Delta t)\right|\left|\tilde{P}_{\mathrm{R}}^{(3)}(\Omega;\Delta t)\right|\cos\Delta\Phi, \qquad \text{(S34)}$$

where $\Delta\Phi$ denotes phase difference between $\tilde{P}_{\mathrm{R}}^{(3)}(\Omega;\Delta t)$ and $\tilde{P}_{\mathrm{NR}}^{(3)}(\Omega;\Delta t)$. While the contribution of the vibrationally resonant $\tilde{P}_{\mathrm{R}}^{(3)}(\Omega;\Delta t)$, which is directly related to $\chi_R^{(3)}(\Omega)$ (Eq. (S26)), is contained in the second term of Eq. (S34), the contribution of the purely resonant signal in the absence of NRB is given by $\left|\tilde{P}_{\mathrm{R}}^{(3)}(\Omega_0;\Delta t)\right|^2$. Therefore, to quantify the coherent enhancement achieved by the interference with the NRB, we defined a coherent amplification factor w_{R} representing the ratio of the interferometrically amplified resonant term

$2\left|\tilde{P}_{\mathrm{NR}}^{(3)}(\Omega;\Delta t)\right|\left|\tilde{P}_{\mathrm{R}}^{(3)}(\Omega;\Delta t)\right|$ to the intrinsic resonant term $\left|\tilde{P}_{\mathrm{R}}^{(3)}(\Omega;\Delta t)\right|^2$ in the absence of NRB at $\Omega = \Omega_0$.

$$\begin{aligned} w_{\mathrm{R}} &\equiv 2\left|\tilde{P}_{\mathrm{NR}}^{(3)}(\Omega_0;\Delta t)\right|\left|\tilde{P}_{\mathrm{R}}^{(3)}(\Omega_0;\Delta t)\right|/\left|\tilde{P}_{\mathrm{R}}^{(3)}(\Omega_0;\Delta t)\right|^2 \\ &= 2\left|\tilde{P}_{\mathrm{NR}}^{(3)}(\Omega_0;\Delta t)\right|/\left|\tilde{P}_{\mathrm{R}}^{(3)}(\Omega_0;\Delta t)\right| \\ &= 2\left|\tilde{P}_{\mathrm{NR}}^{(3)}(\Omega_0;\Delta t)\right|^2/\left|\tilde{P}_{\mathrm{NR}}^{(3)}(\Omega_0;\Delta t)\right|\left|\tilde{P}_{\mathrm{R}}^{(3)}(\Omega_0;\Delta t)\right|. \end{aligned} \quad \text{(S35)}$$

For instance, the amplification factor w_{R} at a probe delay time of Δt = 500 fs can be roughly estimated from the total CARS spectrum shown in the inset of Figure 2(a). In this case, the observed interference amplitude corresponding to the second term of Eq. (S34), $2\left|\tilde{P}_{\mathrm{NR}}^{*(3)}\right|\left|\tilde{P}_{\mathrm{R}}^{(3)}\right|$, is approximately 10 counts/s, while the NRB intensity $\left|\tilde{P}_{\mathrm{NR}}^{(3)}\right|^2$ without the resonant contribution is about 70 counts/s. Then, from Eq. (S35), w_{R} is estimated as $2 \times 70/5 = 28$. At shorter delays (e.g. Δt<< 500 fs), the vibrational signal is coherently amplified by more than two orders of magnitude (w_{R}>10^2). However, due to the overwhelming NRB intensity, $\left|\tilde{P}_{\mathrm{NR}}^{(3)}\right|^2$ > 10^3 counts /s (Figure 2), the weak resonant signal from Ångström-scale thick interfacial molecular system is drowned out by the dominant NRB signal from the metal substrate. These considerations highlight that an appropriate probe delay must be chosen to reduce the contribution of $\left|\tilde{P}_{\mathrm{NR}}^{(3)}\right|$ to a level where it remains sufficiently large to provide interferometric amplification, yet not so large as to obscure the resonant contribution. In the present system, a probe delay of ~500 fs satisfies this condition, maintaining $\left|\tilde{P}_{\mathrm{NR}}^{(3)}\right|$ at a level approximately one order of magnitude larger than $\left|\tilde{P}_{\mathrm{R}}^{(3)}\right|$ (inset of Figure 2(a)), thereby enabling efficient observation and amplification of the vibrational signal through the interference term in Eqs. (S30) and (S34). In practice, we find that delays in the range of approximately 500 ± 100 fs provide a favorable signal-to-background condition in the present system, and we therefore adopt 500 fs as a representative measurement condition (Figures 2-4).

Section 8: Supplementary data

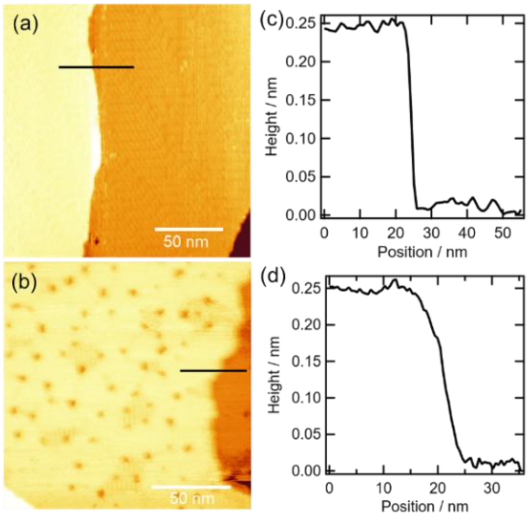

Figure S8. (a, b) Typical scanning tunneling microscopy (STM) image of the samples taken (a) before and (b) after the formation of self-assembled monolayer (SAM) of benzyl mercaptan (BM) molecules on the Au(111) surface. The BM-SAM on Au(111) was prepared by immersing the substrate in an ethanolic solution of BM for 24 hours. These STM images were obtained using an Au tip in constant current mode.[10,27,28] The tunneling current setpoints were 1 nA at -0.5 V for (a) and 0.4 nA at +0.4 V for (b). The substrate prior to immersion exhibited the straight step edges and atomically flat, wide terraces characteristic of clean Au(111). After SAM formation, the step edges became slightly roughened, and patch-like patterns characteristic of the well-defined aromatic thiolate SAMs typically reported in previous STM studies[1,2] appeared on the terraces. Notably, the patch-like patterns in (b) originate from SAMs,[1,2] indicating the formation of a saturated monolayer of BM molecules on Au(111). (c, d) Height profiles taken along the terraces across the step edges indicated by the black lines in the STM images of (a) and (b), respectively.

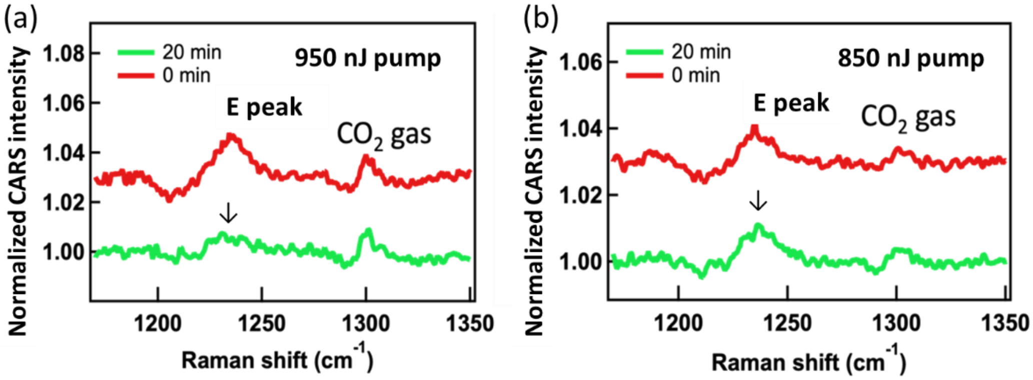

Figure S9. Evaluation of sample damage threshold under varying laser pulse energies. Normalized CARS intensity spectra $I_{\mathrm{CARS}}(\Omega;\Delta t)/I_{\mathrm{NRB}}(\Omega;\Delta t)$ (Eq. (S32)) obtained at different pulse energies of the ω_1 pump pulse (a) 950 nJ (19 mJ/cm^2). (b) 850 nJ (17 mJ/cm^2). The spectra are vertically offset for visual clarity. The pulse energies of the Stokes and probe pulse (Figure S1) were fixed at 100 nJ (2 mJ/cm^2) and 2.7 μJ (54 mJ/cm^2), respectively. Under irradiation with the 950 nJ pump pulse, a gradual decrease in the vibrationally resonant signal from the C-H ring deformation mode of the benzyl mercaptan self-assembled monolayer (SAM) on Au(111) (assigned as the E peak in Figure 4 and S10) was observed over 20 minutes, while the vibrationally resonant signal from the ambient CO_2 gas within the coherence length of the three-beam CARS geometry remained constant, indicating gradual sample damage under the 950 nJ pump pulse. In contrast, no measurable signal attenuations were observed under irradiation the pump pulse below 850 nJ. These results indicate that the pump pulse energies employed in the main text, well below the damage threshold of ~900 nJ, are appropriate for nondestructive CARS measurements for benzyl mercaptan SAM on Au(111).

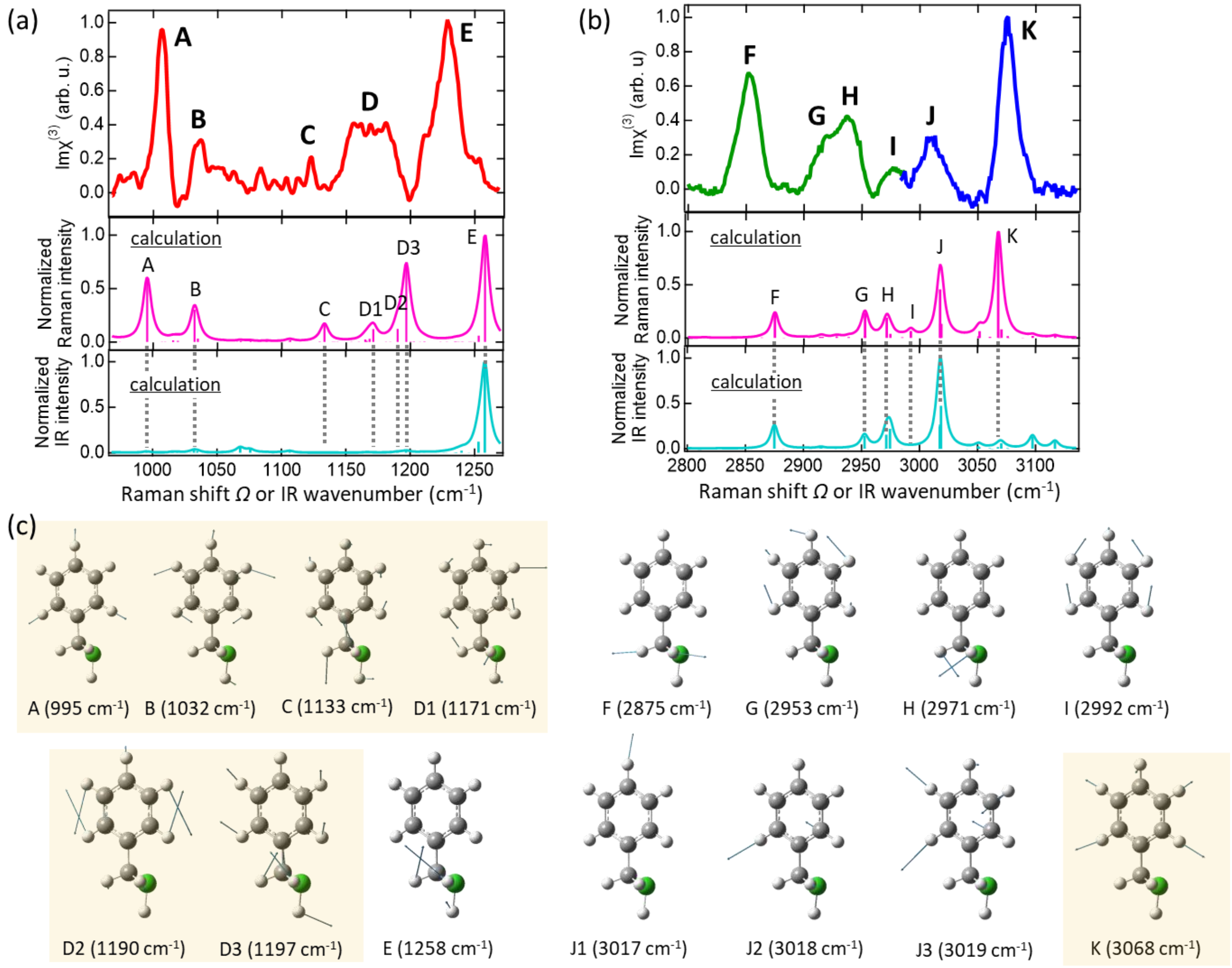

Figure S10. Vibrational spectra of the BM-SAM on Au(111) in (a) the fingerprint region and (b) the C-H stretching region. Top: experimentally retrieved $\mathrm{Im}\chi_R^{(3)}(\Omega)$ spectra. Middle and bottom: Raman intensity and IR absorption spectra of a BM molecule simulated by DFT calculation (see Methods). (c) Representative vibrational motion of Raman-active but IR-inactive modes (A-D, I and K) and Raman- and IR-active modes (E-H and J). Raman-active but infrared-inactive modes include highly symmetric phenyl-ring vibrations such as the ring-breathing (A), ring-bending (B), ring-bending coupled with CH_2 twisting (C), multiple ring-bending coupled with CH_2 twisting or wagging (D), phenyl C-H stretching vibration with minor ring-deformation character (I), and symmetric phenyl C-H stretching (K) modes. In contrast, Raman- and infrared-active modes are assigned as follows: (E) CH_2 wagging, (F), CH_2 symmetric C-H stretching involved in Fermi resonance with a CH_2 bending overtone, (G) phenyl C-H vibration coupled with ring-bending motion, (H) CH_2 bending overtone involved in Fermi resonance with the symmetric CH_2 C-H stretching vibration, and (J) multiple lower-symmetry phenyl C–H stretching modes.

Table S1. Comparison of Raman activities calculated for several molecular species, including homonuclear diatomic molecules and a BM molecule. Vibrational frequencies and Raman activities of H_2, O_2, and N_2 stretching modes and modes A and K of a BM molecule (see Figures 4 and S10) were obtained by using DFT calculations incorporating anharmonic effects (see Section 1 for the details of calculation procedures)

Vibrational mode	Calculated vib. frequency / cm^{-1}	Calculated Raman activity / $Å^6$
H_2 stretching	4164.5	0.31
N_2 stretching	2406.6	0.10
O_2 stretching	1606.3	0.11
Benzylmercaptan mode A	995.2	0.38
Benzylmercaptan mode K	3067.5	1.36

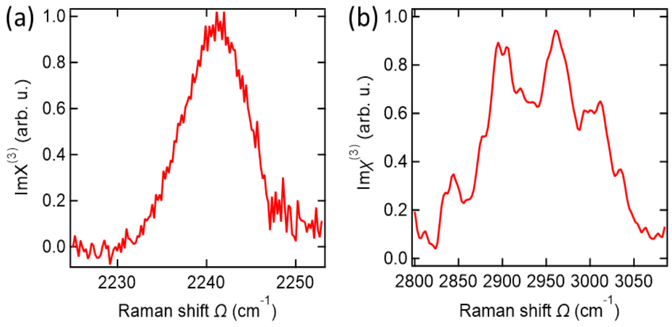

Figure S11. $\mathrm{Im}\chi_R^{(3)}(\Omega)$ CARS spectra of (a) C≡N stretching band of 4-mercaptobenzonitrile and (b) C-H stretching band of dodecanethiol self-assembled monolayers (SAMs) on Au(111), obtained at probe delays of Δt = 1000 fs, and 600 fs respectively, indicating that the present time-frequency hybrid CARS approach is not specific to the BM-SAM shown in Figures 4 and S10. These SAMs were prepared by immersing gold substrates into ethanolic solution of respective molecules for 24 hours.

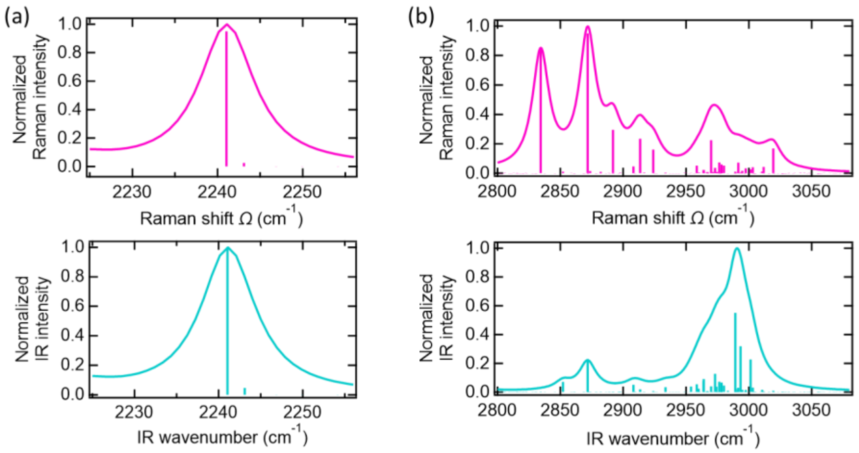

Figure S12. Calculated Raman (upper panel) and infrared (IR) absorption (lower panel) spectra of (a) the C≡N stretching mode of a 4-mercaptobenzonitrile molecule and (b) the C-H stretching band of a hexanethiol molecule. Geometry optimization and vibrational analyses were performed using DFT calculation incorporating anharmonic effects (see Section 1 for details of calculation procedures). To reduce the computational cost while retaining the essential vibrational characteristics of long-chain alkanethiols, hexanethiol was used as a representative model molecule in place of dodecanethiol (Figure S11) for the calculations shown in (b).

REFERENCES

1. Hallmann, L.; Bashir, A.; Strunskus, T.; Adelung, R.; Staemmler, V.; Wöll, C.; Tuczek, F. Self-assembled monolayers of benzylmercaptan and p-cyanobenzylmercaptan on Au(111) surfaces: structural and spectroscopic characterization. *Langmuir* **2008**, *24*, 5726–5733.
2. Hong, M.; Yokota, Y.; Hayazawa, N.; Kazuma, E.; Kim, Y. Homogeneous dispersion of aromatic thiolates in the binary self-assembled monolayer on Au(111) via displacement revealed by tip-enhanced Raman spectroscopy. *J. Phys. Chem. C* **2020**, *124*, 13141–13149.
3. Rajalingam, K.; Hallmann, L.; Strunskus, T.; Bashir, A.; Wöll, C.; Tuczek, F. Self-assembled monolayers of benzylmercaptan and para-cyanobenzylmercaptan on gold: surface infrared spectroscopic characterization. *Phys. Chem. Chem. Phys.* **2010**, *12*, 4390–4399.
4. Moskovits, M. Surface-Enhanced Spectroscopy. *Rev. Mod. Phys.* **1985**, *57,* 783–826.
5. Sugimoto, T.; Matsumoto, Y. Orientational ordering in heteroepitaxial water ice. *Phys. Chem. Chem. Phys.* **2020**, *22*, 16453–16466.
6. Frisch, M. J.; Trucks, G. W.; Schlegel, H. B.; Scuseria, G. E.; Robb, M. A.; Cheeseman, J. R.; Scalmani, G.; Barone, V.; Petersson, G. A.; Nakatsuji, H.; et al. Gaussian 16 Rev. C.01, 2016.
7. V. Barone, Vibrational zero-point energies and thermodynamic functions beyond the harmonic approximation, *J. Chem. Phys.* **2004**, *120*, 3059–3065.
8. V. Barone, Anharmonic vibrational properties by a fully automated second-order perturbative approach, *J. Chem. Phys.* **2005**, *122*, 014108.
9. Dennington, R.; Keith, T. A.; Millam, J. M. GaussView Version 6; Semichem Inc., 2016.
10. Sakurai, A.; Takahashi, S.; Mochizuki, T.; Hirano, T.; Morita, A.; Sugimoto, T. Tip-enhanced sum frequency generation spectroscopy using temporally asymmetric pulse for detecting weak vibrational signals. *J. Chem. Phys.* **2026**, DOI: 10.1063/5.0310824.
11. Miller, J. D.; Slipchenko, M. N.; Meyer, T. R. Probe-pulse optimization for nonresonant suppression in hybrid femtosecond/picosecond coherent anti-Stokes Raman scattering at high temperature. *Opt. Express* **2011**, *19*, 13326–13333.
12. Kumar, V.; Osellame, R.; Ramponi, R.; Cerullo, G.; Marangoni, M. Background-free broadband CARS spectroscopy from a 1-MHz ytterbium laser. *Opt. Express* **2011**, *19*, 15143–15148.
13. Stauffer, H. U.; Miller, J. D.; Roy, S.; Gord, J. R.; Meyer, T. R. Hybrid femtosecond/picosecond rotational coherent anti-Stokes Raman scattering thermometry using a narrowband time-asymmetric probe pulse. *J. Chem. Phys.* **2012**, *136*, 111101.
14. Pestov, D.; Ariunbold, G. O.; Wang, X.; Murawski, R. K.; Sautenkov, V. A.; Sokolov, A. V.; Scully M. O. Coherent versus incoherent Raman scattering, molecular coherence excitation and measurement. *Opt. Lett.* **2007**, *32*, 1725–1727.
15. Cui, M.; Bachler, B. R.; Ogilvie, J. P. Comparing coherent and spontaneous Raman scattering under biological imaging conditions. *Opt. Lett.* **2009**, *34*, 773–775.
16. Prince, B. D.; Chakraborty, A.; Prince, B. M.; Stauffer, H. U. Development of simultaneous frequency- and time-resolved coherent anti-Stokes Raman scattering for ultrafast detection of molecular Raman spectra. *J. Chem. Phys.* **2006**, *125*, 044502.
17. Stauffer, H. U.; Miller, J. D.; Slipchenko, M. N.; Meyer, T. R.; Prince, B. D.; Roy, S.; Gord, J. R. Time- and frequency-dependent model of time-resolved coherent anti-Stokes Raman scattering (CARS) with a picosecond-duration probe pulse. *J. Chem. Phys.* **2014**, *140*, 024316.

18. Wang, K.; Florence, J. T.; Hua, X.; Han, Z.; Shen, Y.; Wang, J.; Wang, X.; Sokolov, A. V. Coherent vibrational anti-Stokes Raman spectroscopy assisted by pulse shaping. *Molecules* **2025**, *30*, 2243.
19. Xu, C.; Zhang, Y.; Feng, Q.; Liang, R.; Tian, C. Self-suppression of the giant coherent anti-Stokes Raman scattering background for detection of buried interfaces with submonolayer sensitivity. *J. Phys. Chem. Lett.* **2022**, *13*, 1465–1472.
20. Zhang, C.; Wang, J.; Jasensky, J.; Chen, Z.-Q. Molecular orientation analysis of alkyl methylene groups from quantitative coherent anti-Stokes Raman scattering spectroscopy. *J. Phys. Chem. Lett.* **2015**, *6*, 1369–1374.
21. Wang, J.; Wloch, G.; Lin, T.; Chen Z. Investigating thin silicone oil films using four-wave mixing spectroscopy and sum frequency generation vibrational spectroscopy. *Langmuir* **2021**, *37*, 14540–14549.
22. Bito, K.; Okuno, M.; Kano, H.; Leproux, P.; Couderc, V.; Hamaguch, Hiro. Three-pulse multiplex coherent anti-Stokes/Stokes Raman scattering (CARS/CSRS) microspectroscopy using a white-light laser source. *Chem. Phys.* **2013**, *419*, 156-162.
23. Wang, X.; Wang, K.; Welch, G. R.; Sokolov, A. V. Heterodyne coherent anti-Stokes Raman scattering by phase control of its intrinsic background. *Phys. Rev. A* **2011**, *84*, 021801.
24. Suzuki, T.; Misawa, K. Efficient heterodyne CARS measurement by combining spectral phase modulation with temporal delay technique. *Opt. Express* **2011**, *19*, 11463–11470.
25. Day, J. P. R.; Domke, K. F.; Rago, G.; Kano, H.; Hamaguchi, H.-o.; Vartiainen, E. M.; Bonn, M. Quantitative coherent anti-Stokes Raman scattering (CARS) microscopy. *J. Phys. Chem. B* **2011**, *115*, 7713–7725.
26. Vartiainen, E. M.; Rinia, H. A.; Müller, M.; Bonn, M. Direct extraction of Raman line-shapes from congested CARS spectra. *Opt. Exp*. **2006**, *14*, 3622-3630.
27. Sakurai, A.; Takahashi, S.; Mochizuki, T.; Sugimoto, T. Tip-Enhanced Sum Frequency Generation for Molecular Vibrational Nanospectroscopy, *Nano Lett.* **2025**, *25,* 6390–6398.
28. Takahashi, S.; Kumagai, K.; Sakurai, A.; Mochizuki, T.; Hirano, T.; Morita, A.; Sugimoto, T. Tip-Enhanced Sum-Frequency Vibrational Nanoscopy beyond the Diffraction Limit, *J. Phys. Chem. C* **2026**, *130,* 373–385.
